# Clinician awareness and implementation of vitamin B_12_ monitoring guidance in metformin users: a primary care survey

**DOI:** 10.1186/s12875-026-03185-w

**Published:** 2026-01-27

**Authors:** Ian Parsonage, David Wainwright, Julian Barratt

**Affiliations:** 1 Department of Health, University of Bath, Bath, UK; 2 School of Psychology, Health and Clinical Sciences, Aston University, Aston, Birmingham, UK

**Keywords:** Vitamin B_12_, Metformin, Deficiency, Knowledge, Screening, Healthcare professional, Clinician, Awareness

## Abstract

**Background:**

Vitamin B_12_ deficiency is a recognised adverse effect of long-term metformin use. In 2022, the United Kingdom (UK) Medicines and Healthcare products Regulatory Agency (MHRA) issued a Drug Safety Update advising clinicians to consider periodic vitamin B_12_ monitoring in at-risk patients. However, no specific testing interval or operational guidance has been published, and uptake within primary care remains uncertain. This study aimed to assess clinician awareness of metformin-associated vitamin B_12_ deficiency, evaluate the impact of the MHRA alert, and identify perceived barriers and facilitators to implementing evidence-based monitoring practices.

**Methods:**

A cross-sectional online survey was distributed to primary care clinicians across the Southwest of England. Eligible participants included registered healthcare professionals involved in Type 2 Diabetes (T2DM) care. The validated BARRIERS scale was used to measure factors influencing evidence use, with internal consistency assessed using Cronbach’s α. Descriptive and inferential statistics, including Chi-squared and Kruskal–Wallis tests, were applied to explore associations between professional role, awareness, and testing behaviour.

**Results:**

A total of 124 clinicians completed the survey (approximate response rate 48%), representing a multidisciplinary primary-care workforce. Reported awareness of the association between metformin and vitamin B_12_ deficiency was high (87%), yet only 35% had read the MHRA alert directly, and 39% reported annual vitamin B_12_ testing. No significant associations were found between awareness, professional role, or testing frequency (*p* > 0.05). The highest mean barrier scores were within the innovation domain, reflecting ambiguity around the clarity and applicability of guidance, while the adopter domain recorded the highest facilitator scores, indicating strong individual motivation when supported by accessible resources.

**Conclusions:**

Clinician knowledge of metformin-related vitamin B_12_ deficiency is high, but behavioural change remains limited by unclear guidance and organisational constraints. Barriers relate predominantly to the characteristics of the innovation and its implementation context rather than individual motivation. Integrating vitamin B_12_ monitoring into existing T2DM review templates and strengthening national guidance could enhance uptake.

**Supplementary Information:**

The online version contains supplementary material available at 10.1186/s12875-026-03185-w.

## Background

More than 12 million people in the UK, equivalent to 1 in 5 adults, now have diabetes or pre-diabetes and that number is only increasing every year. The UK spend £10.7 billion on diabetes care each year, and this is set to rise to £18 billion by 2035[1]. 415 million people live with diabetes worldwide, and an estimated 193 million people have undiagnosed diabetes [1], and its development is primarily caused by a combination of two main factors: defective insulin secretion by pancreatic β-cells and the inability of insulin-sensitive tissues to respond to insulin [2].

Metformin has been used for treating T2DM since the late 1950s. Alongside its antihyperglycemic activity, it was shown to be a potential drug candidate for treating a range of other diseases that include various cancers, cardiovascular diseases, diabetic kidney disease, neurodegenerative diseases, renal diseases, obesity, inflammation, COVID-19 in diabetic patients, and aging [3].

Metformin acts majorly by activating AMPK (Adenosine Monophosphate-Activated Protein Kinase) in the cells and reducing glucose output from the liver [4]. More than 150 million diabetic patients are thought to receive the drug regularly worldwide [5].

Vitamin B_12_, is a water-soluble vitamin, which once ingested, is released from its food carrier proteins by proteolysis in the acidic environment of the stomach [6]. Vitamin B_12_ is an essential micronutrient required especially for optimal hematopoietic and neurological function. Vitamin B_12_ deficiency can cause serious clinical symptoms such as megaloblastic anaemia, paralysis, dementia, fatigue, and mood disturbance. If left untreated, serious neurological and neuropsychiatric complications can occur [7].

In the last 20 years, there was increasing evidence of vitamin B_12_ deficiency among metformin treated diabetic patients [8]. Many studies have reported that vitamin B_12_ deficiency is related to the malabsorption of vitamin B_12_ among metformin-treated T2DM patients [9–11]. Evidence suggests that metformin impairs vitamin B_12_ status primarily in a dose and duration dependent manner.

Several mechanisms have been proposed to explain the effect of metformin on vitamin B_12_; the most widely accepted mechanism is the inhibition of the calcium-dependent binding of the B_12_ intrinsic factor complex to the cubilin receptor in the ileum [12]. The prevalence reported in various studies, of vitamin B_12_ deficiency in metformin-treated patients with diabetes, ranges from approximately 6% to 50%[6].

Although the higher prevalence of vitamin B_12_ deficiency in patients with T2DM receiving metformin therapy is well established, there are no specific guidelines regarding screening modalities for vitamin B_12_ deficiency in this population [13]. Currently, in the UK, the Clinical Knowledge Summary (CKS) lists vitamin B_12_ deficiency as an adverse effect [14].

The CKS was updated after June 2022, in which the MHRA published advice, which concluded that this side effect occurs more frequently than was previously thought [15]. This has led to an update to the product information for all metformin containing medications [13]. The MHRA [15] states that low vitamin B_12_ is now considered to be a common side effect, especially when taking high-dose or long-term metformin, affecting up to one in 10 people [14].

The MHRA [15] advocates periodic monitoring of vitamin B_12_ in patients on metformin with risk factors for B_12_ deficiency. The MHRA [15] advice, reminds practitioners that when a person has anaemia or neuropathy caused by vitamin B_12_ deficiency, it is important that treatment starts as soon as possible to avoid the development of permanent symptoms. Nevertheless, no published national guidelines have been identified regarding a criteria for screening [6].

Awareness of monitoring for adverse effects from medication has been shown to be poor [14]. Additionally, there is evidence consistently demonstrating limited knowledge among primary care clinicians regarding adverse drug reactions (ADRs) [16–18]. Surveys of prescribers show substantial gaps in awareness of serious ADRs associated with commonly prescribed agents such as nitrofurantoin, statins and metformin, where long-term toxicities remain under-recognised by clinicians around the world [16–18].

It is well recognised that publishing guidance does not automatically lead to its use in day-to-day primary care. Programmes, such as the ASPIRE and IMP2ART, demonstrate the difficulty of embedding multiple EBPs into general practice [19, 20]. Subsequently, inconsistencies in ADR knowledge illustrates how limited awareness of the evidence translates into varying patient safety practices.

A recent published example, of this is shown through a recent MHRA’s update on nitrofurantoin hepatotoxicity and how it translates into routine GP consultations. The study indicated that there was poor awareness of front-line clinicians with the monitoring recommendations [21, 22].

Pharmacovigilance studies have shown that ADR under-reporting is derived from a combination of low confidence, perceived complexity of reporting systems, and lack of time in primary care consultations [23]. These barriers mirror the wider literature on EBP implementation, which identifies workload, knowledge deficits, and competing clinical priorities as key barriers [20, 24].

There are currently no published studies exploring UK primary care clinicians’ awareness of the risk of vitamin B_12_ deficiency in long-term metformin use [14]. The aim of this study is therefore twofold: first, to assess clinicians’ awareness of the association between long-term metformin therapy and vitamin B_12_ deficiency, and to examine whether awareness has changed following the recent MHRA alert [15]; and second, to explore clinicians’ perceptions of the key barriers and facilitators to implementing evidence-based practice in primary care.

## Method

### Study design

This quantitative study employed a cross-sectional survey design, using an anonymised online questionnaire to examine the clinician awareness (self-reported) of the association between metformin and vitamin B_12_ deficiency, and awareness of the MHRA alert [15]. The survey was distributed to registered healthcare professionals working in primary care settings across the Southwest of England who were involved in the management of patients with T2DM. The questionnaire incorporated the validated BARRIERS scale [25] to assess perceived barriers and facilitators to evidence-based practice.

### Study setting

In the NHS footprint of the Southwest region there are 7 Integrated Care Boards (ICBs) 83 Primary Care Networks (PCNs) which represent a total of 559 GP practices. The Southwest region was chosen as it represents practices which are embedded in urban areas, costal and rural areas, reflecting key organisational and workload characteristics of UK primary care and supporting cautious transferability of the findings. The costs and practicalities of conducting this research project means the author must restrict the sample to a realistic size.

Table 1 demonstrates the inclusion/exclusion criteria for this study. The participants do not necessarily have to be prescribers, as many practice nurses with specialist interest undertake patients’ diabetic reviews.

**Table 1:** Inclusion criteria

Inclusion criteria
Registered healthcare professional in the UK
Work predominantly in general practice in the NHS footprint of the Southwest of England
Involved in the clinical review of patients with T2DM who are being treated with metformin

### Study participants and sampling

The sample was recruited through reaching out to GP practices across differing geographical areas within the Southwest, that represent both rural and urban practices. The individual clinician is the unit of measurement.

NHS Digital [24] has measurements of the number of registered GPs, nurses, and allied health professionals in GP practices in 2023. According to NHS Digital [26] in the South-West NHS geographical region there are 3855 GPs, 2172 registered nurses (this includes Practice Nurses, Advanced Nurse Practitioners etc.) and 401 (registered other healthcare professionals (paramedics and pharmacists) who would fit the inclusion criteria of this study.

**Fig. 1:**
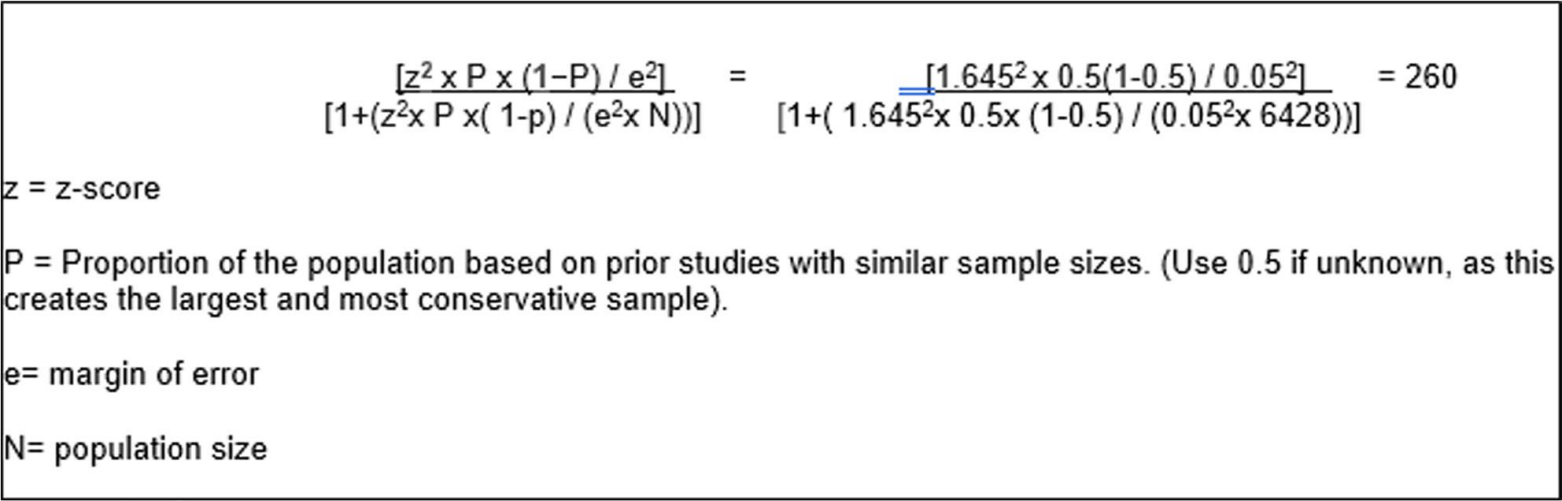
Sample size equation

Based on a population size of 6428, with a confidence level of 90% and a margin of error of 5%, a sample size of 260 participants would need to participate. A 90% confidence level was chosen to balance statistical precision with the practical constraints of clinician recruitment in primary care. See Fig. 1 for sample size calculation.

Stratified sampling was employed to help recruit potential participants. The strata of this sample were each of the seven ICBs areas within the South-West geographical region. From each stratum 4 practices were selected using random sampling and all staff who fit the inclusion criteria within the surgery will be invited to take part. An email was sent to the chosen generic practice email address, with each practice asked to forward the study invitation to all clinicians involved in the care of patients with type 2 diabetes. This approach may have introduced selection bias, as practices with more organised administrative systems or higher engagement with clinical governance activities may have been more likely to disseminate the invitation internally. A reminder email was sent approximately two months after the initial invitation in an attempt to improve response rates across practices.

### Participants’ recruitment

The email provided included the study information and questionnaire link and was asked to be forwarded to all relevant clinicians within the GP practices which are part of eligible to participate (GPs, Nurses and Allied Healthcare professionals who are involved in the care of patients with T2DM). The survey was sent and open for completion from May 2025-September 2025.

To improve recruitment to low response rates from the initial practices identified, other practices from the low response areas were contacted via their generic NHS email address, directly inviting them and their appropriate staff to participate.

### Data collection

The questionnaire was accessed by an electronic link sent via the email. Implied consent was assumed by participants submitting the questionnaire. The questionnaire was made up of closed ended questions. Closed ended questions was followed by a Likert scale response, or a discrete option, in order for data analysis to occur. Responses were anonymised to ensure confidentiality and encourage candid responses. The survey was self-developed for the purpose of this study and has not been validated.

The questionnaire used the BARRIERS scale [25] was developed to assess clinicians’, administrators’, and academicians’ perceptions of barriers to the use of research findings in practice. The scale items were developed from literature on research utilisation, the Conduct and Utilisation of Research in Nursing (CURN) project questionnaire, and data gathered from nurses [25]. The reliability of the scale has been assessed through various studies and the Cronbach’s alfa of the BARRIERS scale [27] was 0.94, with the test-retest value 0.60 [27, 28]. No permission was required to use this scale.

The ‘JISC’ online platform hosted the online questionnaire.

### Data analysis

Summary statistics were used to describe responses for each item within the barriers and facilitators elements of the questionnaire. Mean values, standard deviations, and response frequencies were calculated to summarise the extent to which each factor was perceived to influence the use of evidence-based practice. Internal reliability for each scale was assessed using Cronbach’s α, with coefficients exceeding 0.9 indicating excellent internal consistency across items.

Inferential statistics were applied to explore associations between selected variables. For categorical or non-parametric data, the Chi-squared test was used to examine relationships between awareness or behavioural variables, for example, the relationship between awareness of the MHRA alert [15] and professional role or prescriber status. The Kruskal–Wallis test was employed to compare ordinal responses between professional groups (GP, nurse, pharmacist, paramedic).

Normality of continuous and ordinal variables was assessed using the Shapiro–Wilk test (Shapiro–Wilk W = 0.957, *p* < 0.001). As the total BARRIERS score deviated significantly from a normal distribution, non-parametric tests were used for subsequent analyses. Therefore, to explore associations between the overall barrier and facilitator scores and indicators of evidence-based behaviour, Spearman’s rank-order correlation (ρ) was applied.

Specifically, correlations were tested between total barrier scores and the reported frequency of clinical guideline use, and between total facilitator scores and the self-reported frequency of vitamin B_12_ testing. Internal consistency of the BARRIERS barrier and facilitator scales within this sample was assessed using Cronbach’s α as a descriptive measure of scale coherence. This was undertaken to confirm that the items functioned consistently within this dataset and not as a formal psychometric validation of the instrument.

A two-tailed significance level of *p* < 0.05 was considered indicative of statistical significance.

## Results

A total of 124 clinicians completed the survey, representing a broad mix of primary care professionals. This equates to an estimated 48% of the intended sample. The exact response rate could not be determined as the total number of clinicians who received or viewed the invitation is unknown due to distribution via generic practice email addresses. The 124 participants represent the final analytical sample. Of those that responded, most were prescribers actively involved in diabetes care or long-term condition management (see Table 2).

**Table 2:** Describing the characteristic statistics of the respondents completing the questionnaire

Characteristic	*n* (%)
Total participants (n)	124 (100%)
GP	15 (12%)
Nurse	94 (76%)
Paramedic	4 (3%)
Pharmacist	11 (9%)
Prescriber	41 (33%)
Type of care for diabetic patients delivered -	
Acute	23 (19%)
Routine care	29 (23%)
Diabetic review	96 (77%)
Medication review	36 (29%)

Awareness of the association between metformin and vitamin B_12_ deficiency was reported high amongst the respondents (108 (87%)). This result is further supported by 40% (49) of the respondents stating that they have read the MHRA alert [15] for metformin whilst 42% of the respondents (52) had not read the alert but have seen the link or reference to the alert in other publications (see Table 3).

**Table 3:** Showing the responses from the questionnaire related the MHRA alert [15]

MHRA alert	*n* (%)
Total participants (n)	124 (100%)
Aware of the complications may be associated with long-term (> 6months) Metformin use?	108 (87%)
Aware of link between metformin and vitamin B_12_ deficiency	107 (86%)
Aware of MHRA alert (2022)	
Yes	49 (40%)
Not of the alert but have seen the link in other published articles	52 (42%)
Has read the MHRA alert	
Yes	43 (35%)
Not directly, but aware of it through research paper/article	51 (41%)
MHRA alert altered their practice	
Yes	43 (35%)
I was already monitoring for this	46 (37%)
Received formal education on B _12_ deficiency risk	9 (7%)
Believe guidelines for metformin use are sufficient/clear	45 (36%)

Identification of the clinical symptoms of vitamin B_12_ deficiency varied. While the majority recognised fatigue and anaemia correctly (123 (99%) and 114 (92%)), this reduced when identifying peripheral neuropathy or cognitive as a potential presentation (84 (68%) and 78 (63%).

Routine monitoring practices were also variable. Almost all respondents reported reviewing for side effects of metformin on either a 6 monthly (40 (32%)) or yearly (74 (60%)) basis. However, 48 (39%) respondents check vitamin B_12_ levels in the bloods on a yearly basis and 68 (55%) of respondents check for vitamin B_12_ levels in the blood if the patient is felt to be symptomatic.

Only 41 (33%) viewed prescribers as primarily responsible for ongoing monitoring, and 45 (36%) considered the available guidance clear. Additionally, only 48 (39%) respondents felt that there were readily available resources or guidelines in their practice setting to aid them. Furthermore, 72 (58%) of respondents felt that their organisation offers sufficient support to stay up to date with the MHRA medication safety alerts.

The relationships between awareness of the MHRA alert [15], professional role, and clinical behaviour were examined using Chi-squared and Kruskal–Wallis tests. Awareness of metformin-related vitamin B_12_ deficiency did not differ significantly by profession (χ² 6.20, p 0.10), nor did awareness of the MHRA alert [15] differ significantly by prescriber status (χ² 5.32, p 0.07). See Table 4.

**Table 4:** Association between clinician characteristics and awareness, testing behaviour, and opinion using χ² and Kruskal–Wallis tests

Group	Test	Statistic	Degrees of freedom (df)	*p*-value	Interpretation
Awareness of B_12_ deficiency in Metformin vs. Profession	Chi^2^	X^2^ = 6.20	3.0	0.102	No significant difference
Awareness of MHRA alert vs. Prescriber status	Chi^2^	X^2^ = 5.32	2.0	0.070	Borderline-near set P value 0.05 (prescribers slightly more aware)
Frequency of B_12_ testing vs. awareness	Kruskal–Wallis	H = 0.12	-	0.729	No significant difference

Testing frequency was not significantly different between those aware and unaware of the association of vitamin B_12_ deficiency and long-term use of metformin (H 0.12, p 0.73). While differences did not reach statistical significance on Kruskal–Wallis testing, mean rank scores (see Table 4) showed that prescribers and those aware of the MHRA alert [15] reported somewhat higher testing frequency and greater confidence in identifying vitamin B_12_ deficiency symptoms.

The most commonly reported barriers to implementing evidence-based practice were lack of time and difficulty accessing research. “No time to read research” and “research reports or articles are not readily available” were reported to a moderate or greater extent by most respondents (see Table 5). Other frequently reported barriers included the large volume of available information, uncertainty regarding where to locate relevant evidence, and limited access to clear summaries at the point of care.

**Table 5:** Top 5 barriers and facilitators identified in implementing evidenced based practice

Rank	Barrier	Reported to moderate/great extent (%)	Facilitator	Reported to moderate/great extent (%)
**1**	No time to read the research	88 (71%)	Training/Education days	115 (93%)
**2**	Research reports/articles are not readily available	85 (68%)	Clinical Knowledge Summaries/NICE	111 (89%)
**3**	Insufficient time on the job to implement new ideas	79 (64%)	Local formulary	108 (87%)
**4**	Amount of research information is overwhelming	75 (61%)	Clinical websites	107 (87%)
**5**	Relevant literature is not compiled in one place	69 (56%)	Clinical decision tools embedded in the electronic notes	101 (82%)

Facilitators most often identified included access to education and concise, authoritative guidance. The majority of respondents reported that training and education sessions, NICE CKS, and local formulary or practice protocols supported their ability to apply evidence in daily practice (see Table 5). Access to professional networks, peer discussion, and clinical websites were also described as factors that made evidence easier to locate and use.

When the individual barrier and facilitator items were grouped according to the four domains described by Funk et al.[25] innovation, communication, organisation, and adopter, the highest mean barrier scores were recorded within the innovation domain. This domain included items relating to the clarity, relevance, and practical applicability of guidance. The adopter domain, representing individual knowledge, confidence, and motivation, showed the lowest mean barrier values across all respondents.

For facilitators, the highest mean scores were within the adopter domain. This was followed by innovation and communication domain. Organisational facilitators, such as protected time or managerial support, recorded the lowest mean scores overall. The mean values and ranking of each domain are presented in Table 6.

**Table 6:** Mean barrier and facilitator scores grouped by domain using the funk et al. [23] framework

Domain	Barrier Mean ± SD	Rank	Facilitator Mean ± SD	Rank
Innovation	2.38 ± 0.77	1	3.45 ± 0.56	2
Communication	2.09 ± 0.83	2	3.14 ± 0.55	3
Organisation	2.08 ± 0.67	3	2.57 ± 0.61	4
Adopter	1.45 ± 0.76	4	3.56 ± 0.61	1

Internal consistency of the BARRIERS scale within this sample was assessed using Cronbach’s α. The barrier scale yielded an α of 0.969 and the facilitator scale an α of 0.910.

## Discussion

### Awareness-practice gap in vitamin B_12_ monitoring

Though this study identified high levels of awareness of the association between metformin and vitamin B_12_ deficiency (87%), this did not translate into consistent engagement with the MHRA alert [15] or routine monitoring behaviour. Only one-third of respondents had read the MHRA Drug Safety Update [15] directly, and fewer than 40% reported annual vitamin B_12_ testing. Furthermore, neither professional role nor prescriber status was significantly associated with awareness or testing frequency, indicating that the observed variation in practice is unlikely to reflect differences in training, scope of practice, or seniority.

Whilst the differences were non-significant, this likely reflects an implementation issue rather than genuine uniformity across groups. The absence of statistical association is unlikely to represent actual equivalence. It could suggest that awareness of the guidance remains superficial and does not translate into the behavioural change required for routine monitoring.

Furthermore, the lack of differences between roles suggests the problem may be system-level rather than individual. If educational background or prescriber status were the primary contributing factor of guideline adoption, one would expect significant between-group variation [29].

Nevertheless, the observed directional tendency towards higher awareness among prescribers suggests that exposure to MHRA communications and direct prescribing responsibility may modestly increase engagement with the alert. However, the survey data indicate that this effect is insufficient to produce consistent changes in vitamin B_12_ testing behaviour, reinforcing the conclusion that structural and organisational factors outweigh individual-level influences.

The data presented challenge the assumption that increasing awareness alone will drive improvement. The combination of high awareness across staff groups and minimal change in monitoring behaviour implies that the limiting factor lies in how guidance is operationalised. In this respect, the findings quantify the “implementation plateau” described in studies of other medication safety alerts, in which dissemination of guidance leads to limited and short-lived behavioural change in the absence of supporting systems [30].

An implementation plateau is a pattern observed in implementation research in which early improvements in the uptake of evidence or practice level off or regress when active implementation support diminishes or persistent contextual barriers remain [31].

### Why awareness does not translate into monitoring

One contributing factor may be the clinical nature of vitamin B_12_ deficiency itself. Unlike many adverse drug reactions, metformin-associated vitamin B_12_ deficiency develops gradually and produces non-specific symptoms such as fatigue, neuropathy, and cognitive change, which overlap substantially with diabetes and ageing [6, 10, 32]. As a result, deficiency may not trigger reactive investigation in routine consultations, making reliance on opportunistic detection ineffective and increasing the importance of structured, preventive monitoring.

The wording of the MHRA guidance [15] is also likely to be influential. Although the MHRA Drug Safety Update [15] reclassified vitamin B_12_ deficiency as a common adverse effect of metformin, it advises clinicians only to “consider periodic monitoring” without defining frequency, responsibility, or thresholds for action [15], [33]. In the absence of clear operational direction, clinicians must interpret the alert individually, leading to variable and often limited changes in practice. Comparable implementation failures have been reported for other MHRA safety alerts, including those relating to nitrofurantoin pulmonary and hepatic toxicity, where national warnings have not translated into consistent monitoring in primary care [21, 22].

This pattern mirrors findings from previous studies of vitamin B_12_ monitoring in metformin-treated populations. Alshammari et al.[18] reported that although 44% of clinicians were aware of American Diabetes Association [34] recommendations for vitamin B_12_ screening, fewer than 5% of patients had ever been tested. Similarly, Longo et al. [32] and Martin et al. [35] found that awareness of guidance did not result in timely or consistent testing, even among more senior clinicians. Together with the present findings, this suggests that knowledge alone is a weak driver of behaviour in the absence of clear operational pathways.

Evidence from systematic reviews of regulatory safety communications indicates that safety-related regulatory actions often produce variable and limited changes in clinical practice, with less than half of interrupted time-series evaluations reporting meaningful impacts on provider behaviour [36]. This underscores that regulatory alerts alone may be insufficient to drive consistent practice change without additional implementation support.

### Barriers and facilitators to implementation

Using the BARRIERS framework[25], the findings suggest that the main barriers stem less from clinician motivation and more from the clarity and usability of the guidance. The highest mean barrier scores were observed within the innovation domain, indicating that uncertainty regarding the clarity, relevance, and practical applicability of the MHRA recommendations, particularly the absence of defined monitoring intervals, was a key obstacle to implementation. This is consistent with Funk’s original observation that *“research findings which are not presented in an understandable or applicable form will not be utilised, however sound their scientific base” [25].

The absence of significant between-group differences further supports the view that these barriers are systemic rather than individual. Kajermo et al. [27] similarly concluded that organisational and presentation-level barriers are more influential than adopter-level factors in determining evidence use. In contrast, the low adopter-level barrier scores and high facilitator scores observed in this study demonstrate that clinicians are motivated and confident when supported by appropriate resources.

### Implications for UK primary care

Organisational context further shapes behaviour. Respondents commonly reported limited time, workload pressures, and difficulty accessing concise, practice-ready guidance, consistent with wider implementation research in UK primary care [20, 23, 27]. Unlike HbA1c, renal function, or lipid monitoring, vitamin B_12_ testing is not embedded within routine T2DM review templates or incentivised through national quality frameworks, which may further reduce its prioritisation despite acknowledged risk [32, 35].In such settings, guidance that is not integrated into existing workflows is unlikely to be enacted consistently.

Ultimately, these findings demonstrate the persistence of the knowledge–practice gap described in implementation research, in which awareness of evidence does not automatically result in its use. To address this, primary care must move from passive awareness to *“organised readiness”, [25] embedding vitamin B12 monitoring guidance within the routine framework of T2DM chronic disease reviews.

### Strengths and limitations

This study provides new insight into how primary care clinicians interpret and implement the MHRA’s vitamin B_12_ monitoring guidance for metformin users. The use of a validated framework [25] strengthens the methodological rigour, with excellent internal consistency across domains (Cronbach’s α > 0.9). Participation from a broad range of registered healthcare professionals who work in primary care reflects the multidisciplinary nature of T2DM care and supports the reliability of the findings within the primary care setting.

However, a limitation of this study is the reliance on self-reported data, which leads to the possibility of social desirability bias. Through asking clinicians directly whether they are aware of the metformin–vitamin B_12_ association is likely to produce different responses compared with asking them to list the adverse effects of metformin unprompted. This may mean the survey overestimates true underlying knowledge.

Furthermore, the survey used for this study was self-developed for the purpose of this study and not validated. This may have an impact on reliability and generalisability of the results.

Additionally, the survey response rate was modest (124 from a target of 260), and the true denominator cannot be confirmed as distribution through generic practice email accounts prevented tracking. This limits precise calculation of response rate and may introduce response bias. As the sample was confined to clinicians in the Southwest of England, generalisability to other regions should be interpreted with caution.

## Conclusion

This study highlights that while clinician knowledge and awareness of the association between metformin and vitamin B_12_ deficiency is high, translation of that into consistent monitoring practice remains limited. The lack of statistically significant variations between professional groups reinforces that this is a system-level challenge, rooted in how guidance is operationalised rather than a deficit in individual knowledge. The findings suggest that the MHRA alert [15], has a high level of awareness. Yet, it lacks sufficient clarity and incorporation within chronic disease management frameworks to drive meaningful behavioural change.

Connecting this persistent knowledge–practice gap will require embedding vitamin B_12_ monitoring within existing diabetes review processes. This could be further, supported by clearer national guidance, electronic prompts, and dedicated implementation support. In doing so, primary care chronic review systems can move from passive awareness to active implementation, ensuring that safety alerts translate into sustained clinical behaviour change.

### Implications for practice and policy

Improving implementation of vitamin B_12_ monitoring guidance will require clearer operational direction and better integration within existing T2DM disease review processes. Embedding vitamin B_12_ monitoring within routine T2DM review templates, supported by electronic prompts and recall mechanisms in GP clinical systems, would reduce reliance on individual clinician recall and better align monitoring with existing workflows. At a national level, MHRA safety guidance would benefit from greater operational clarity, including specification of monitoring frequency, responsibility and thresholds for action, supported by implementation-ready guidance for use within NICE CKS and primary care electronic records.

A future study could use semi-structured interviews to explore in depth how clinicians interpret and apply MHRA recommendations in practice, and to identify the contextual and organisational factors that influence behaviour. Insights from these interviews will inform targeted strategies to strengthen evidence-based prescribing and safety monitoring across primary care.

## Supplementary Information

Supplementary Material 1.

Supplementary Material 2.

Supplementary Material 3.

## Data Availability

Data is provided within the manuscript or supplementary information files.

## Abbreviations

ADA: American Diabetes Association
ADR: Adverse Drug Reaction
AMPK: Adenosine Monophosphate–Activated Protein Kinase
BARRIERS: Barriers to Research Utilization Scale
CKS: Clinical Knowledge Summary
COVID: 19–Coronavirus Disease 2019
CPD: Continuing Professional Development
CURN: Conduct and Utilisation of Research in Nursing
EBP: Evidence–Based Practice
GP: General Practitioner
HRA: Health Research Authority
ICB: Integrated Care Board
IRAS: Integrated Research Application System
MHRA: Medicines and Healthcare products Regulatory Agency
NHS: National Health Service
NICE: National Institute for Health and Care Excellence
PCN: Primary Care Network
SD: Standard Deviation
T2DM: Type 2 Diabetes Mellitus
UK: United Kingdom
