# Supplementary material for: Clinician awareness and implementation of vitamin B12 monitoring guidance in metformin users: a primary care survey
Source: BMC Prim Care. 2026 Jan 27;27:65. doi: 10.1186/s12875-026-03185-w (PMC12918417; doi:10.1186/s12875-026-03185-w)
Supplement: Supplementary file 1 — Supplementary Material 1. [file 12875_2026_3185_MOESM1_ESM.pdf]

UNIVERSITY OF BATH

Vitamin B12

Awareness of Vitamin B12 deficiency in long term use of Metformin

This questionnaire is produced by Ian Parsonage

IRAS No. 336490

I would like to invite you to take part in a research project. This project is part of a Doctoral student project with the University of Bath, who is also the sponsor of this research

This phase of the study will be exploring if the MHRA alert released in June 2022, regarding the risk of Vitamin B_{12} deficiency in people on metformin has altered clinical practice in primary care. This phase of the study will also be exploring what are the barriers and facilitators in using evidence-based practice in your clinical setting.

The overall aim of the study is to explore the awareness of this alert amongst primary care clinicians and the effectiveness of translating evidence-based practice into clinical practice using this alert as a case study.

This questionnaire contains 24 questions and should take less than 10 minutes.

Your honest responses are vital for improvement of patient care guidelines. Please answer candidly. All answers are anonymous.

Context

What is you profession? ✱ *Required*

- GP
- Nurse
- Paramedic
- Pharmacist
- Physiotherapist
- Physician Associate
- Podiatrist
- Other

What is your primary location of work? ✱ *Required*

- GP surgery
- Out of Hours service
- Urgent Care Centre
- Emergency Department
- Community care i.e. Community Matron, District Nurse etc.
- Other

What type of care do you provide to patients who have type 2 diabetes? ✱ *Required*

- ☐ Acute care (patients who attend with an acute problem, who happen to have type 2 diabetes)
- ☐ Routine care (patients who attend with a concern directly linked to their type 2 diabetes)
- ☐ Diabetes review (patients who attend for their annual type 2 diabetes review)

☐ Medication review (undertake a medication review which includes diabetic medication)

Are you a prescriber? ✱ *Required*

◯ Yes
◯ No

Metformin

Prior to prescribing long-term (> 6 months) Metformin, which of the following baseline checks would you normally complete? ✱ *Required*

- [ ] HbA1c
- [ ] U+Es (renal function)
- [ ] LFTs
- [ ] Bone profile
- [ ] Folate
- [ ] Vitamin B12
- [ ] Ferritin
- [ ] Cholesterol
- [ ] Random Glucose
- [ ] Thyroid Function
- [ ] Height
- [ ] Weight
- [ ] Blood pressure
- [ ] Pulse
- [ ] None

I am aware of the complications may be associated with long-term (> 6 months) Metformin use? ✱ *Required*

- ○ Yes
- ○ No
- ○ Unsure

I believe that the prescriber of Metformin is responsible for monitoring the drug side

effects/complications ✱ *Required*

- Strongly disagree
- Disagree
- Neutral
- Agree
- Strongly agree

I review patients on long-term (> 6 months) Metformin for side effects at an approximate frequency of ✱ *Required*

- Monthly
- 3 monthly
- 6 monthly
- Yearly
- Not at all

Vitamin B12

Are you aware of the signs and symptoms of low vitamin B12 levels? Tick the boxes you feel are related to signs or symptoms of a low Vitamin B12 ✱ *Required*

- ☐ Fatigue
- ☐ Anemia
- ☐ Numbness or tingling in extremities
- ☐ Cognitive changes (e.g., memory loss)
- ☐ Glossitis (inflamed tongue)
- ☐ Muscle weakness
- ☐ Visual disturbances/changes
- ☐ Mood changes (e.g., depression)
- ☐ Thirst
- ☐ Rash
- ☐ Vomiting
- ☐ Urinary frequency
- ☐ Chest pain

How frequently do you assess vitamin B12 levels in patients taking metformin? ✱ *Required*

- ○ Monthly
- ○ 3 monthly
- ○ 6 monthly
- ○ Yearly
- ○ Every 2 years
- ○ Only if patient is symptomatic
- ○ Not at all

Are you aware that metformin has been associated with decreased vitamin B12 levels in some patients? ✱ *Required*

- Yes
- No
- Unsure

Clinical guidance

Are you aware of the MHRA alert regarding the association between long-term metformin use and low vitamin B12 levels? ✱ *Required*

- Yes
- No
- Not of the alert but have seen the link in other published articles/sources

Have you read the MHRA alert regarding Vitamin B_{12} deficiency in long term metformin use? ✱ *Required*

- Yes
- No
- Not directly, but aware of it through research paper/article/colleague

Has the MHRA alert altered your practice in monitoring for vitamin B12 deficiency in long term metformin users? ✱ *Required*

- Yes
- No
- I was already monitoring for this
- I am unaware of the alert

Implementation of evidence

In your opinion, are the current guidelines for long-term (> 6 months) Metformin use sufficient and clear? ✱ *Required*

- Yes
- No
- Unsure

Have you received formal education or training on the potential risk of low vitamin B12 in metformin-treated patients during your medical education or professional development? ✱ *Required*

- Yes
- No
- Unsure

Are there readily available resources or guidelines in your practice setting that address the monitoring and management of vitamin B12 levels in metformin-treated patients? ✱ *Required*

- Yes
- No
- Unsure

In your practice setting, do you feel that there is sufficient organisational support and resources for staying updated on medication safety alerts like the MHRA alert? ✱ *Required*

- Yes
- No
- Unsure

Did your organisation make you aware of the research findings related to vitamin B12 deficiency and metformin use? ✱ *Required*

- Yes
- No
- Unsure

Evidence based practice

How often do you actively seek out or receive updates on research findings and guidelines? ✱ *Required*

- Every day
- Weekly
- Monthly
- 3 monthly
- 6 monthly
- Yearly
- Never

Using the scale below please rate how much each of these factors are a barrier to you using Evidence Based Practice in your clinical practice using the MHRA alert for Vitamin B12 deficiency in Metformin as an example ✱ *Required*

Please don't select more than 1 answer(s) per row.

Please select at least 29 answer(s).

	to no extent	to a little extent	to a moderate extent	to a great extent	No opinion
The research reports/articles are not readily available	☐	☐	☐	☐	☐
The implications for practice are not made clear	☐	☐	☐	☐	☐
The statistical analyses are not understandable	☐	☐	☐	☐	☐

The research is not relevant to the my practice	☐	☐	☐	☐	☐
I am unaware of research	☐	☐	☐	☐	☐
There is a lack of facilities to implement the research	☐	☐	☐	☐	☐
I do not have time to read research	☐	☐	☐	☐	☐
The research has not been replicated	☐	☐	☐	☐	☐
I feel the benefit of changing practice will be minimal	☐	☐	☐	☐	☐
I am uncertain whether to believe the results of the research	☐	☐	☐	☐	☐
The research has methodological inadequacies	☐	☐	☐	☐	☐
The relevant literature is not compiled in one place	☐	☐	☐	☐	☐
I do not feel I have enough authority to change patient care procedures	☐	☐	☐	☐	☐
I feel the results are not generalisable to my own setting	☐	☐	☐	☐	☐

I feel isolated from knowledgeable colleagues with whom to discuss the research	☐	☐	☐	☐	☐
I see little benefit for myself	☐	☐	☐	☐	☐
Research reports/articles are not published fast enough	☐	☐	☐	☐	☐
Colleagues will not cooperate with implementation	☐	☐	☐	☐	☐
Management will not allow implementation	☐	☐	☐	☐	☐
I do not see the value of research for practice	☐	☐	☐	☐	☐
There is not a documented need to change practice	☐	☐	☐	☐	☐
The conclusions drawn from the research are not justified	☐	☐	☐	☐	☐
The literature reports conflicting results	☐	☐	☐	☐	☐
The research is not reported clearly and readably	☐	☐	☐	☐	☐

Other staff are not supportive of implementation	☐	☐	☐	☐	☐
The nurse is unwilling to change/try new ideas	☐	☐	☐	☐	☐
The amount of research information is overwhelming	☐	☐	☐	☐	☐
I do not feel capable of evaluating the quality of the research	☐	☐	☐	☐	☐
There is insufficient time on the job to implement new ideas	☐	☐	☐	☐	☐

Overall in your opinion how significant are the overall barriers to utilising evidence based practice in your clinical setting? ✱ *Required*

Please don't select more than 1 answer(s) per row.

Please select at least 1 answer(s).

	1	2	3	4	
Not significant at all	☐	☐	☐	☐	Very significant

Using the list below rank what you perceive are the factors that help you use research to inform your clinical practice ✱ *Required*

Please don't select more than 1 answer(s) per row.

Please select at least 19 answer(s).

	to no extent	to a little extent	to a moderate extent	to a great extent	no opinion
Training/Education days	☐	☐	☐	☐	☐
Meetings	☐	☐	☐	☐	☐
Supervision	☐	☐	☐	☐	☐
Journal club	☐	☐	☐	☐	☐
Social Media	☐	☐	☐	☐	☐
Clinical Knowledge Summaries/NICE	☐	☐	☐	☐	☐
Journals	☐	☐	☐	☐	☐
Clinical websites	☐	☐	☐	☐	☐
Colleagues	☐	☐	☐	☐	☐
Internal audit	☐	☐	☐	☐	☐
Organisational intranet	☐	☐	☐	☐	☐
Local formulary	☐	☐	☐	☐	☐
Management/Leadership	☐	☐	☐	☐	☐
Time in role/job	☐	☐	☐	☐	☐
Clinical decision tools embedded in the electronic notes	☐	☐	☐	☐	☐
Appraisal	☐	☐	☐	☐	☐
Policies	☐	☐	☐	☐	☐
Internet search	☐	☐	☐	☐	☐
Resources to utilise the evidence	☐	☐	☐	☐	☐

Overall in your opinion how significant are the overall facilitators to utilising evidence based practice in your clinical setting? ✱ *Required*

Please don't select more than 1 answer(s) per row.

Please select at least 1 answer(s).

	1	2	3	4	
Not significant at all	☐	☐	☐	☐	Very significant

Final page

Thank you for taking the time to complete this survey. Your response has been recorded.

If you are interested in taking part in a **online semi-structured interview** exploring this topic further please email ibp25@bath.ac.uk to register your interest.

If you would like to be kept informed with the outcome of this study or require any furtehr information please email ibp25@bath.ac.uk

---
