# Supplementary material for: Clinician awareness and implementation of vitamin B12 monitoring guidance in metformin users: a primary care survey
Source: BMC Prim Care. 2026 Jan 27;27:65. doi: 10.1186/s12875-026-03185-w (PMC12918417; doi:10.1186/s12875-026-03185-w)
Supplement: Supplementary file 3 — Supplementary Material 3. [file 12875_2026_3185_MOESM3_ESM.pdf]

# Vitamin B12

## Awareness of Vitamin B12 deficiency in long term use of Metformin

### Context

1. What is you profession?

Responses: 124

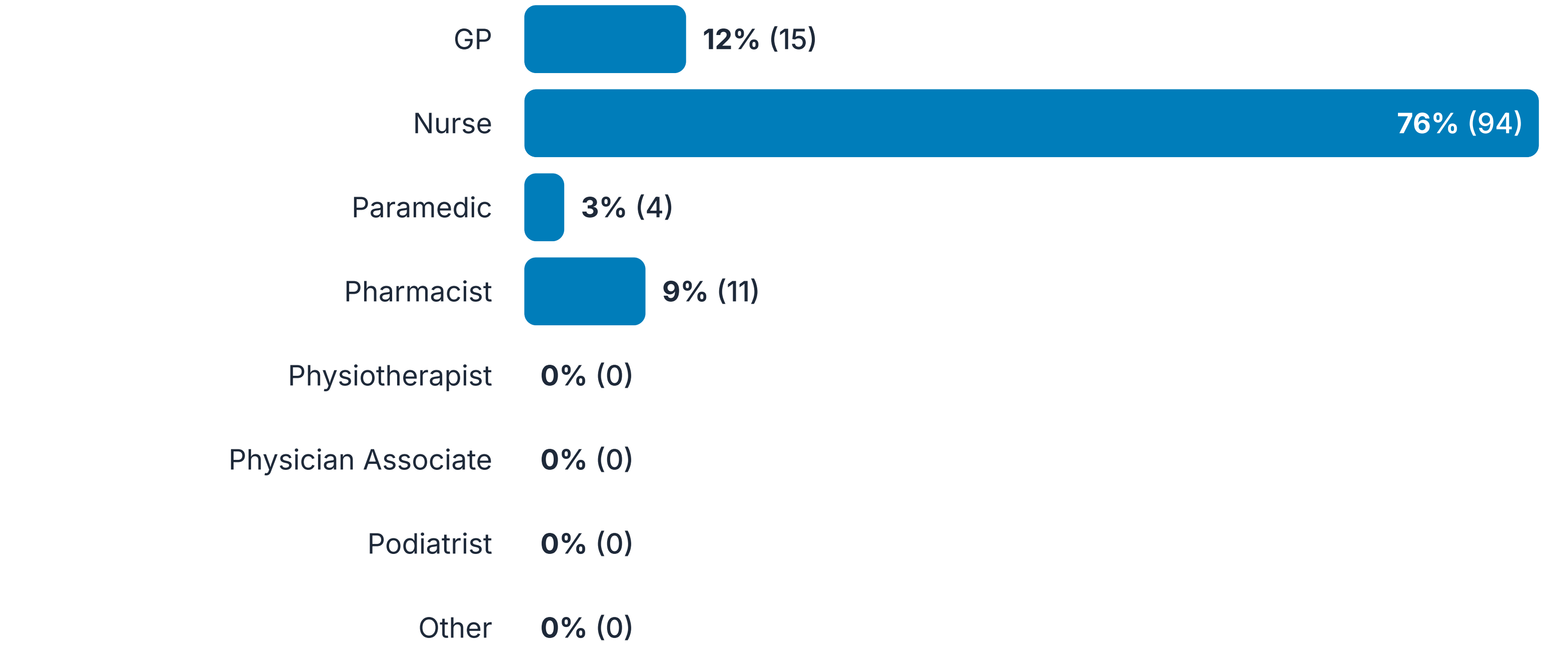


---

2. What type of care do you provide to patients who have type 2 diabetes?

Responses: 124

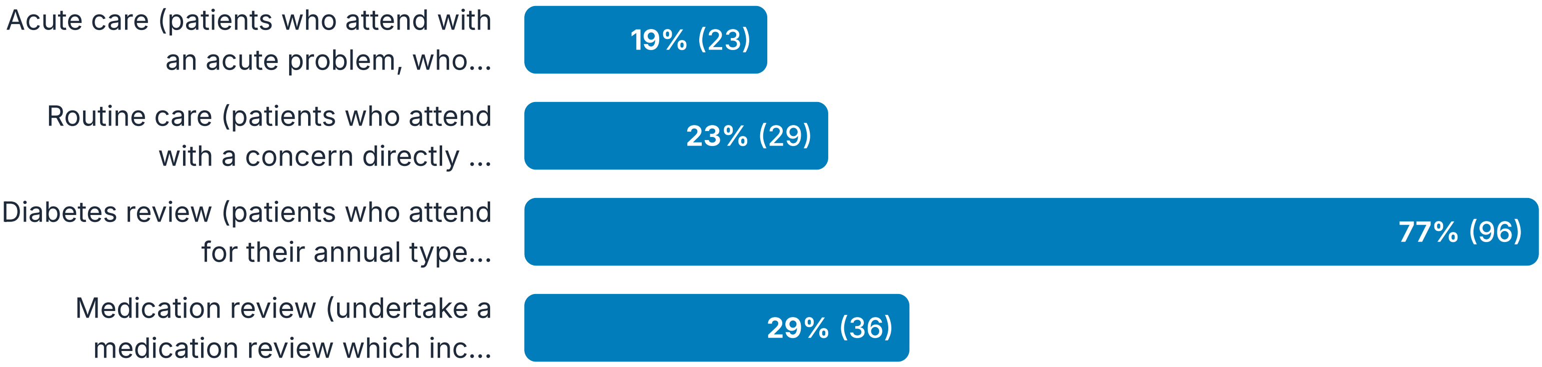


---

3. Are you a prescriber?

Responses: 124

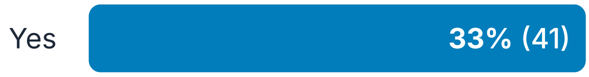

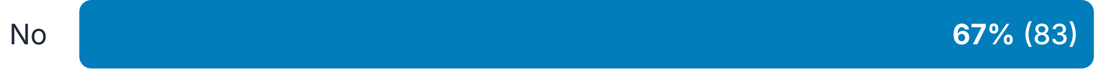


## Metformin

4. Prior to prescribing long-term (> 6 months) Metformin, which of the following baseline checks would you normally complete?

Responses: 124

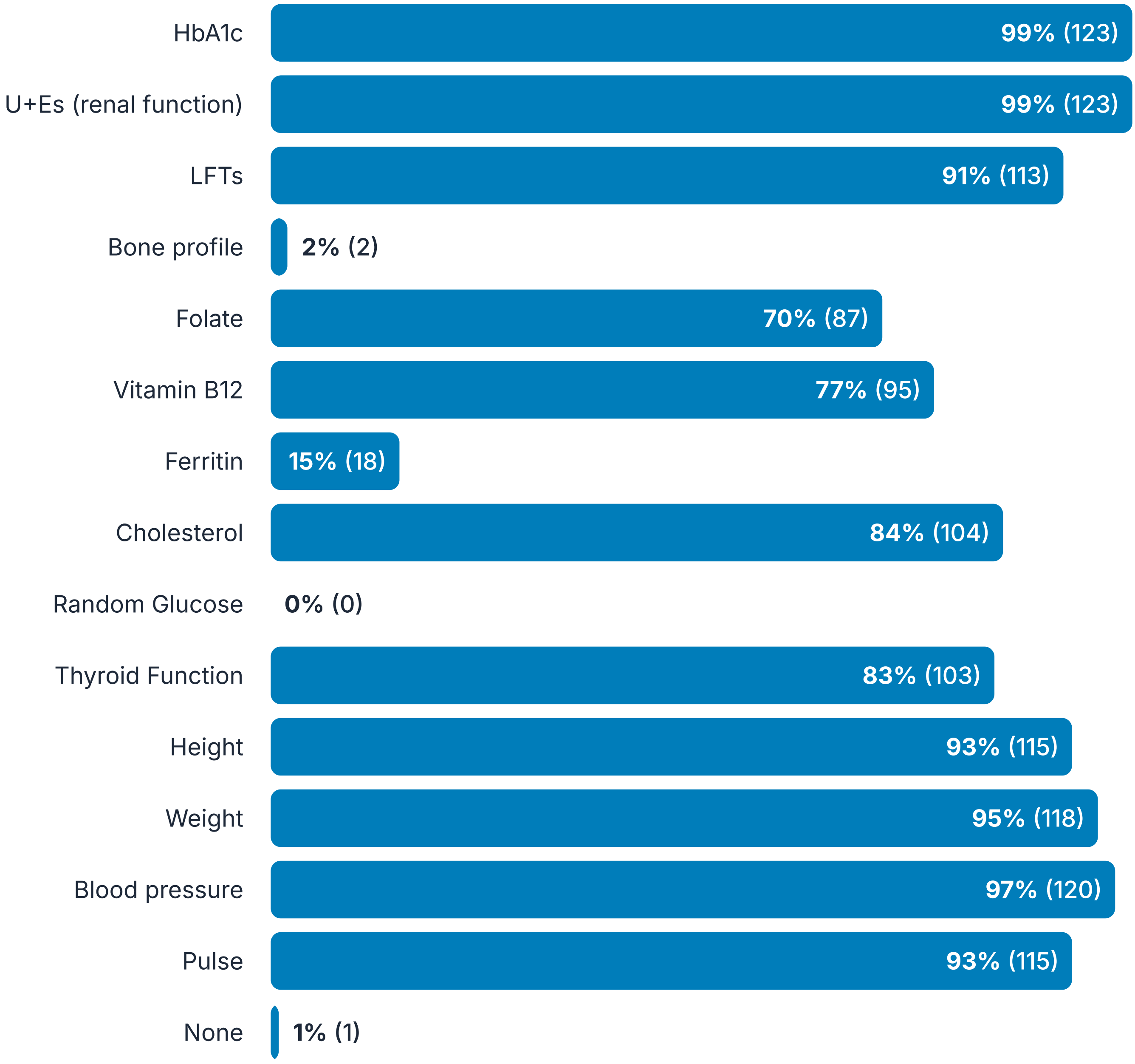


5. I am aware of the complications may be associated with long-term (> 6 months) Metformin use?

Responses: 124

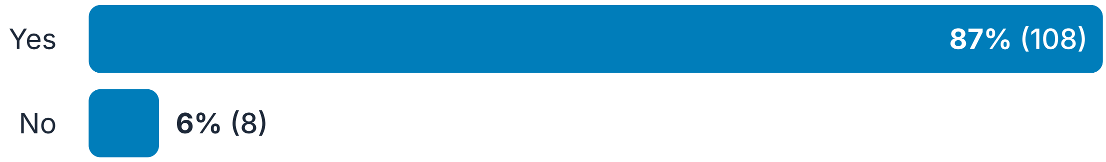

| | |
|---|---|
| Unsure | 6% (8) |

6. I believe that the prescriber of Metformin is responsible for monitoring the drug side effects/complications

Responses: 124

| | |
|---|---|
| Strongly disagree | 18% (22) |
| Disagree | 30% (37) |
| Neutral | 19% (24) |
| Agree | 22% (27) |
| Strongly agree | 11% (14) |

7. I review patients on long-term (> 6 months) Metformin for side effects at an approximate frequency of

Responses: 124

| | |
|---|---|
| Monthly | 1% (1) |
| 3 monthly | 1% (1) |
| 6 monthly | 32% (40) |
| Yearly | 60% (74) |
| Not at all | 6% (8) |

## Vitamin B12

8. Are you aware of the signs and symptoms of low vitamin B12 levels? Tick the boxes you feel are related to signs or symptoms of a low Vitamin B12

Responses: 124

| | |
|---|---|
| Fatigue | 99% (123) |
| Anemia | 92% (114) |
| Numbness or tingling in extremities | 68% (84) |

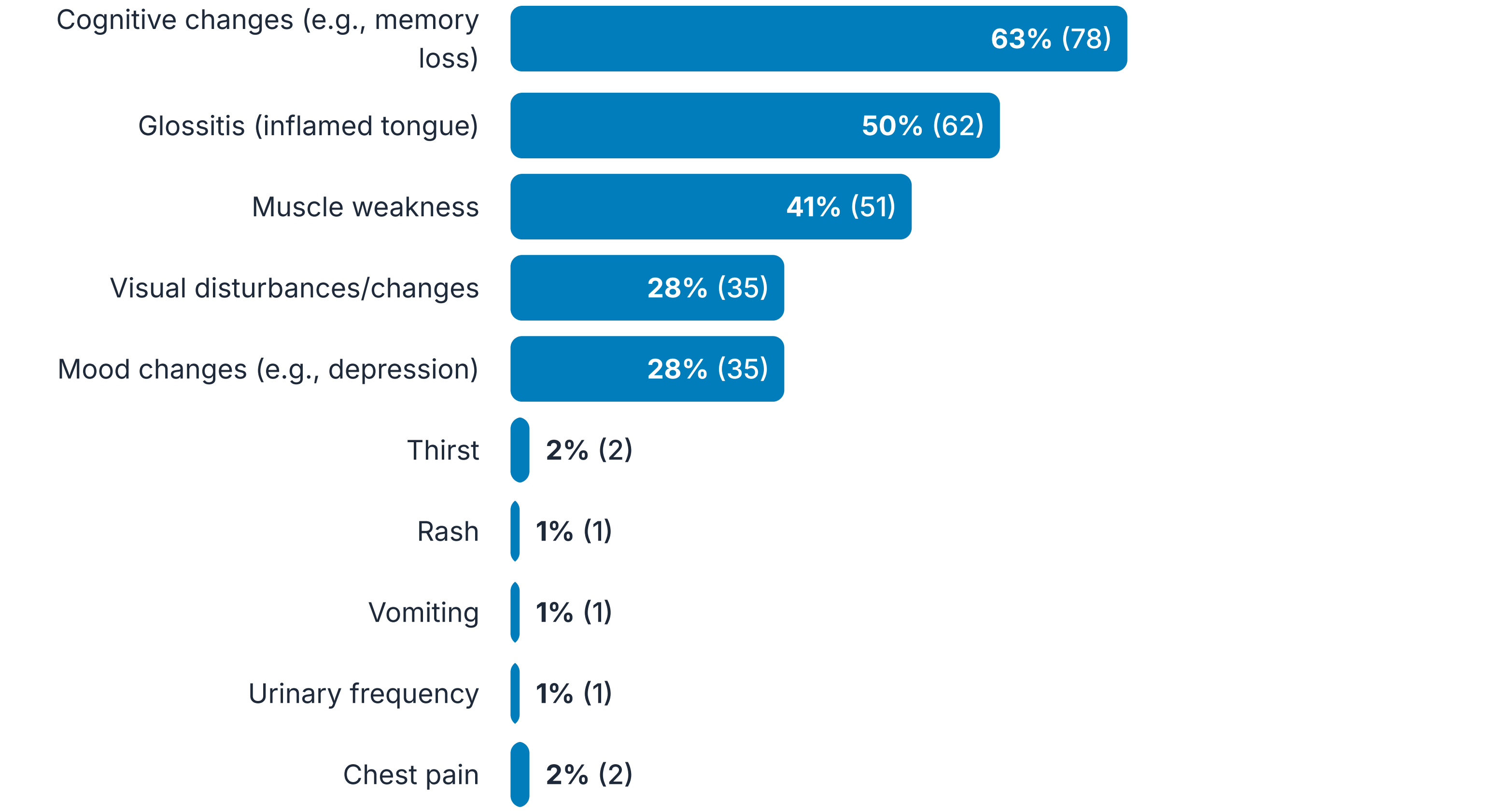


9. How frequently do you assess vitamin B12 levels in patients taking metformin?

Responses: 124

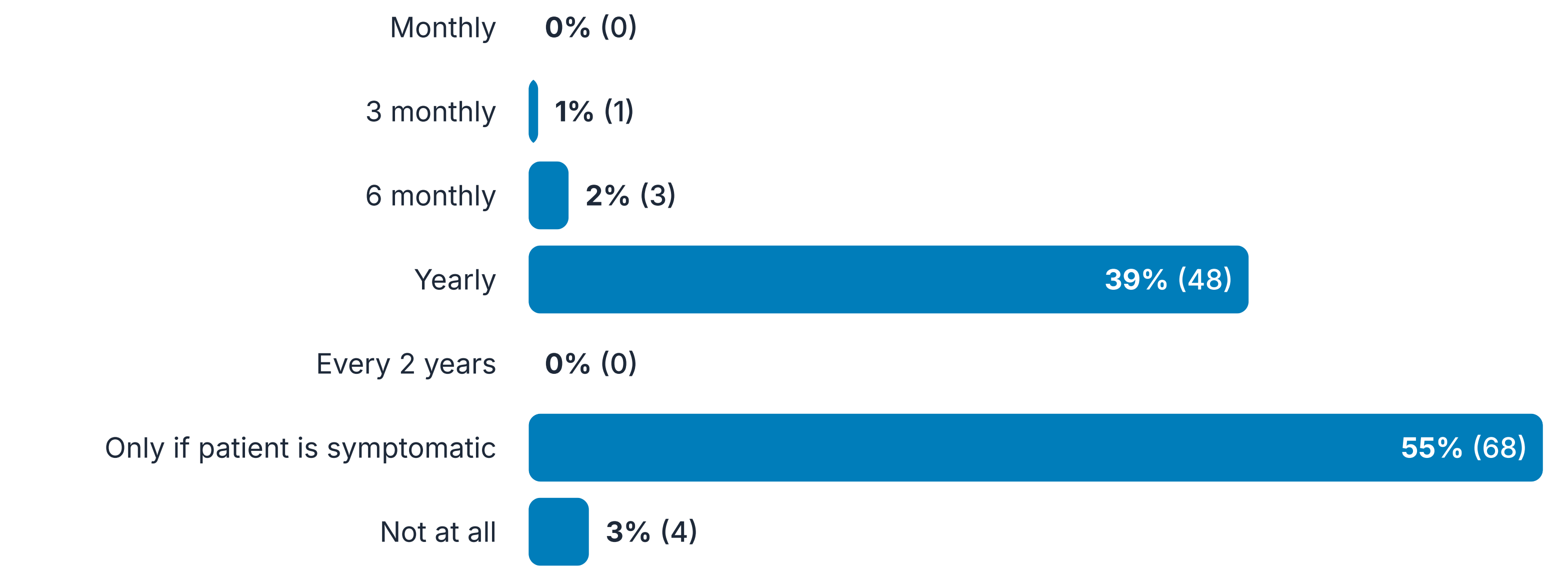


10. Are you aware that metformin has been associated with decreased vitamin B12 levels in some patients?

Responses: 124

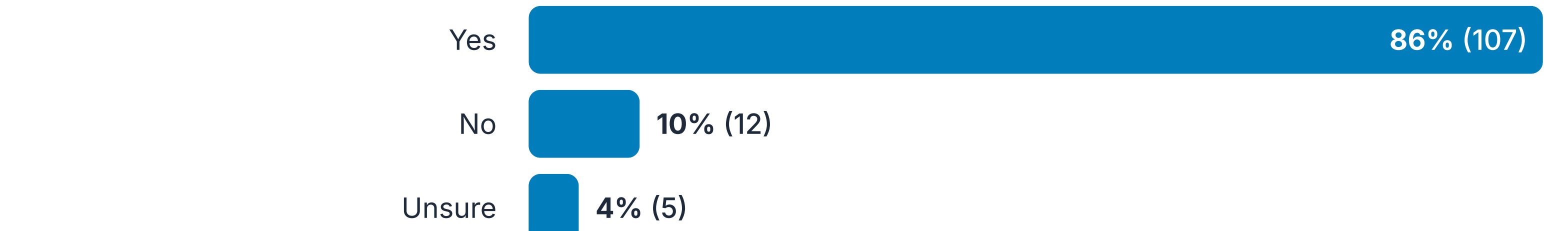

## Clinical guidance

11. Are you aware of the MHRA alert regarding the association between long-term metformin use and low vitamin B12 levels?

Responses: 124

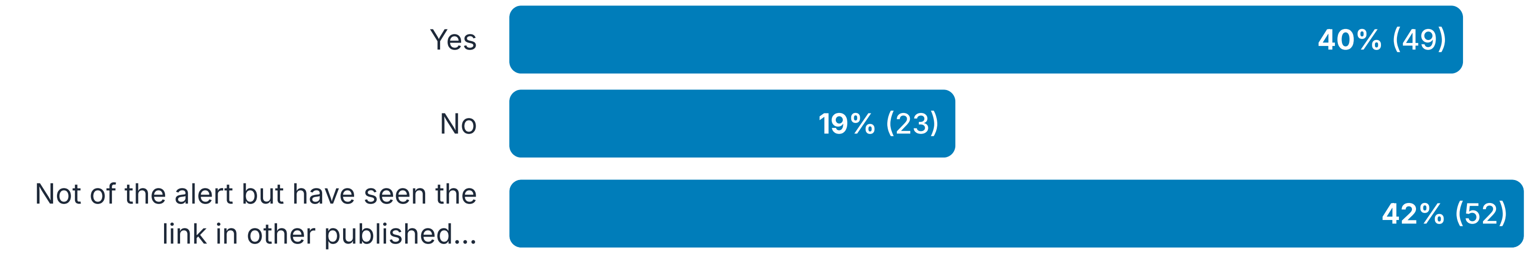


12. Have you read the MHRA alert regarding Vitamin B12 deficiency in long term metformin use?

Responses: 124

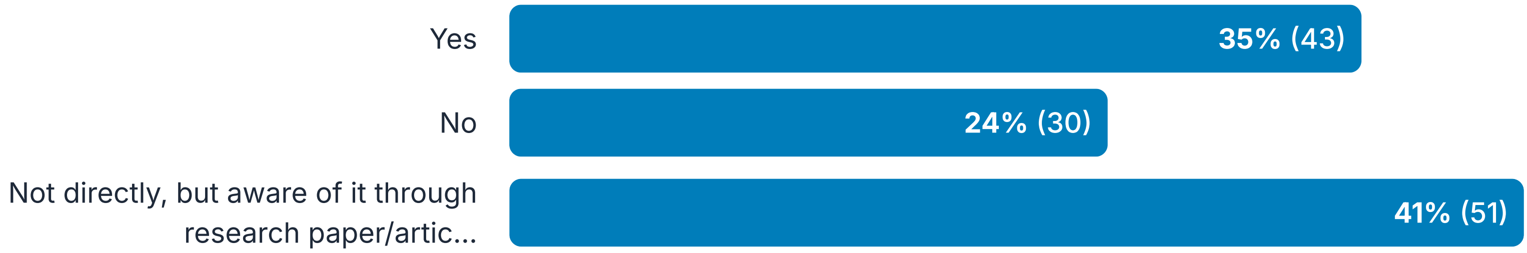


13. Has the MHRA alert altered your practice in monitoring for vitamin B12 deficiency in long term metformin users?

Responses: 124

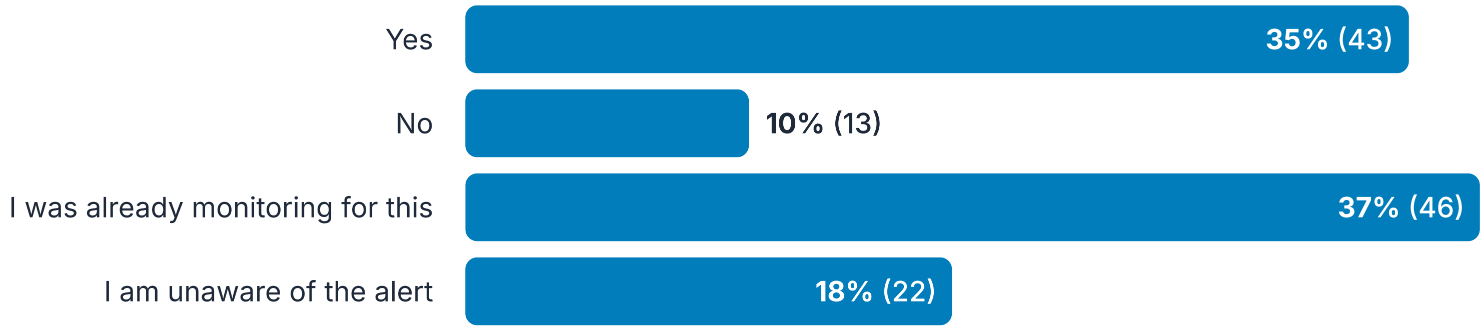


## Implementation of evidence

14. In your opinion, are the current guidelines for long-term (> 6 months) Metformin use sufficient and clear?

Responses: 124

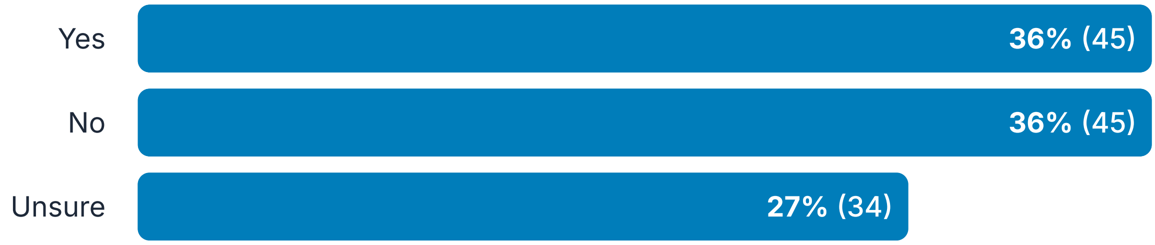


15. Have you received formal education or training on the potential risk of low vitamin B12 in metformin-treated patients during your medical education or professional development?

Responses: 124

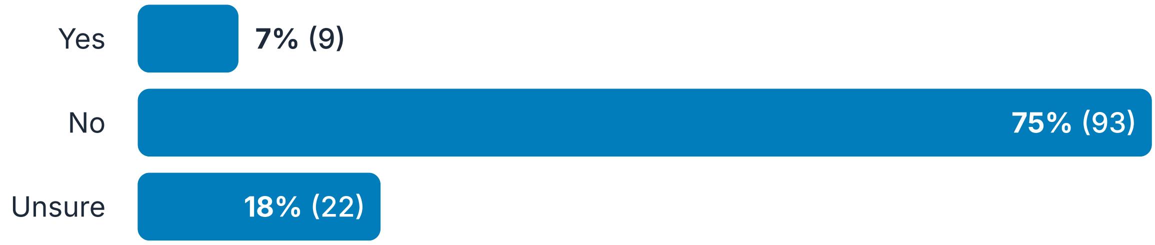


16. Are there readily available resources or guidelines in your practice setting that address the monitoring and management of vitamin B12 levels in metformin-treated patients?

Responses: 124

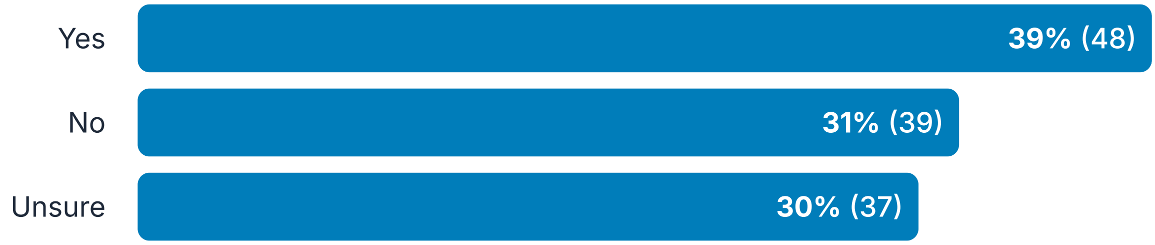


17. In your practice setting, do you feel that there is sufficient organisational support and resources for staying updated on medication safety alerts like the MHRA alert?

Responses: 124

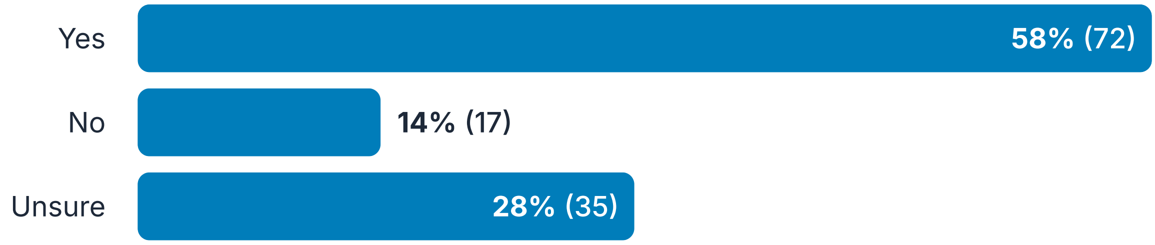

18. Did your organisation make you aware of the research findings related to vitamin B12 deficiency and metformin use?

Responses: 124

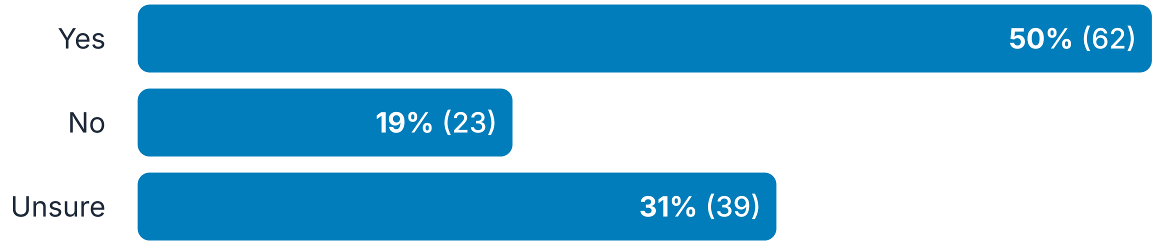


## Evidence based practice

19. How often do you actively seek out or receive updates on research findings and guidelines?

Responses: 124

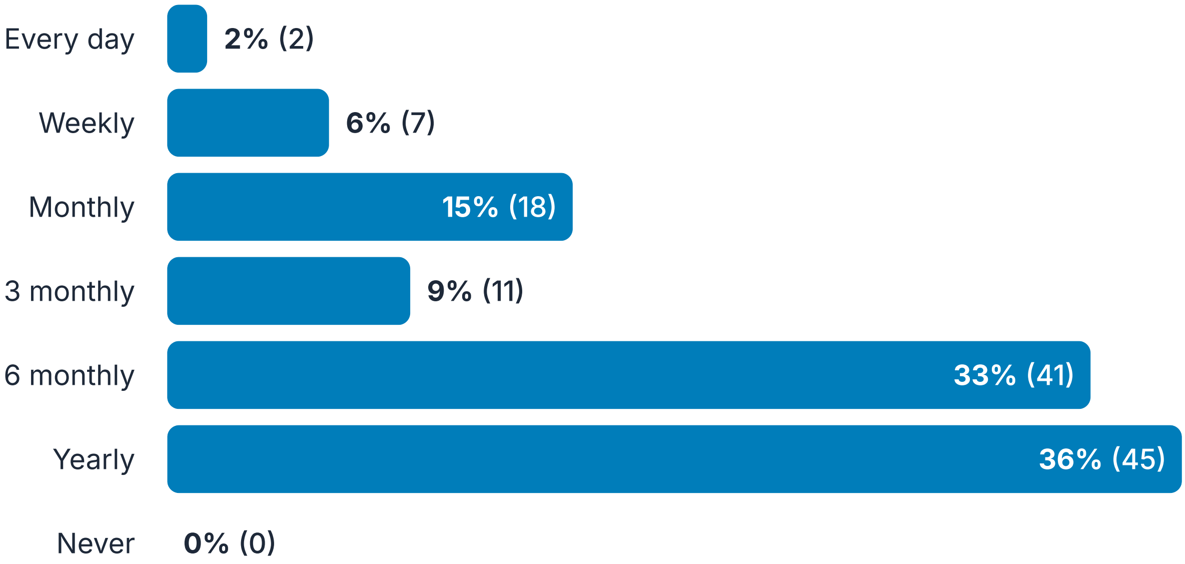


---

20. Using the scale below please rate how much each of these factors are a barrier to you using Evidence Based Practice in your clinical practice using the MHRA alert for Vitamin B12 deficiency in Metformin as an example

Responses: 124

The research reports/articles are not readily available

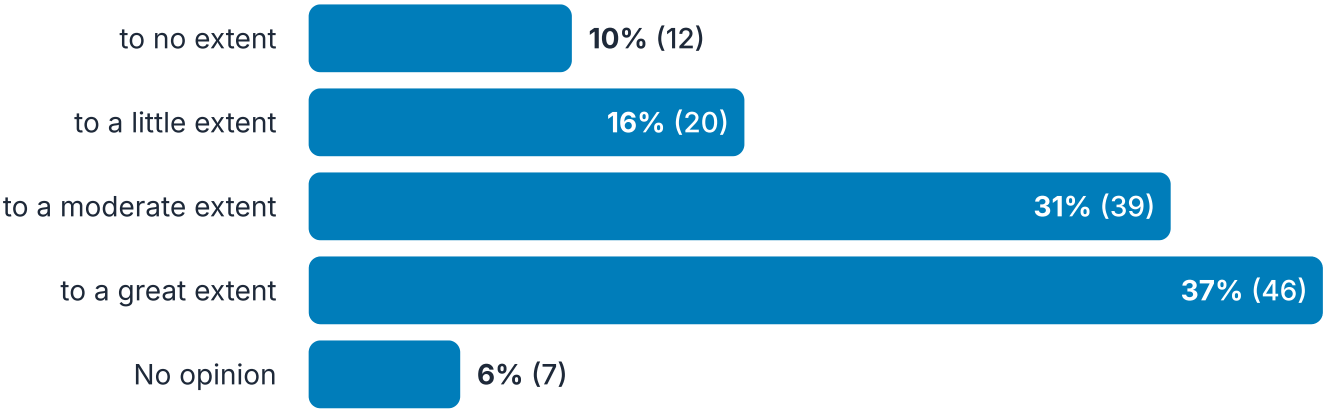

The implications for practice are not made clear

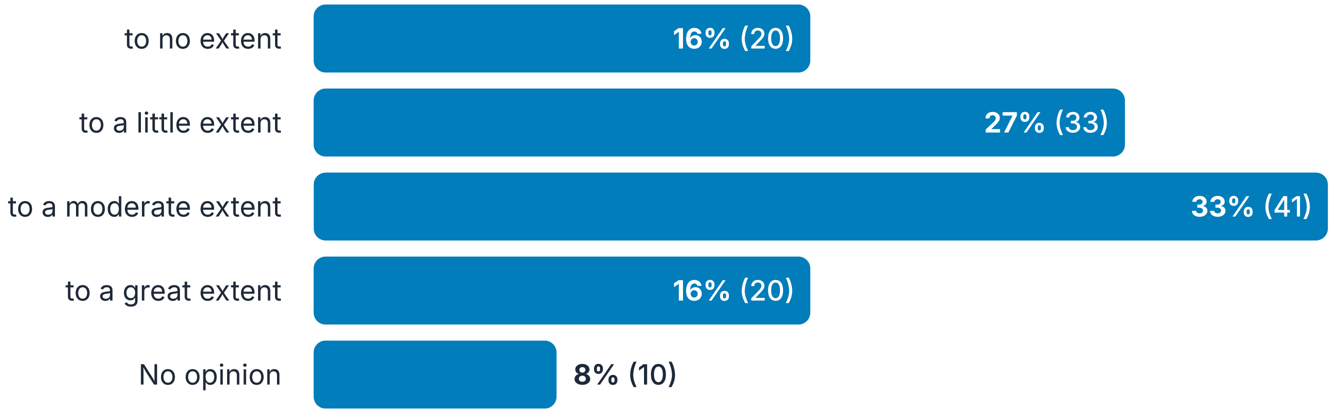


The statistical analyses are not understandable

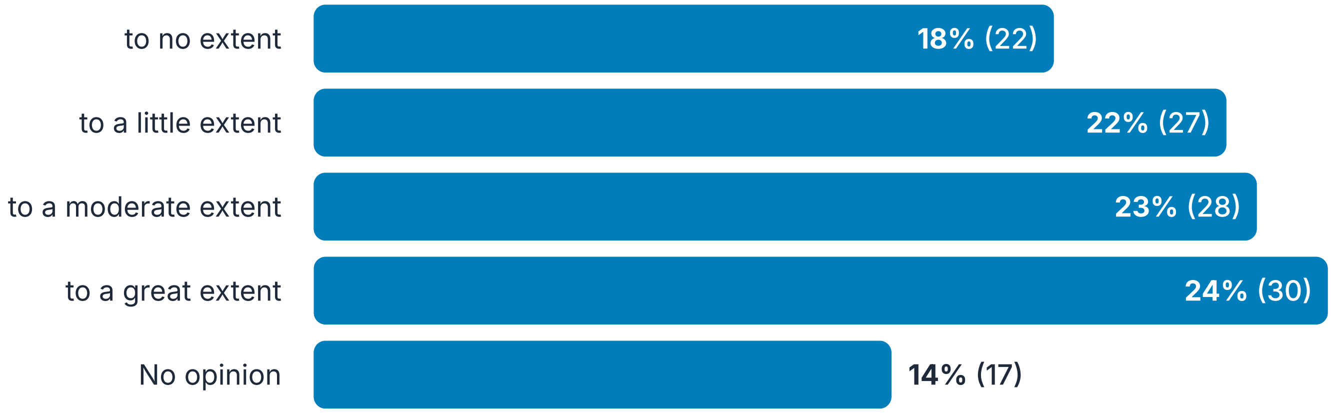


The research is not relevant to the my practice

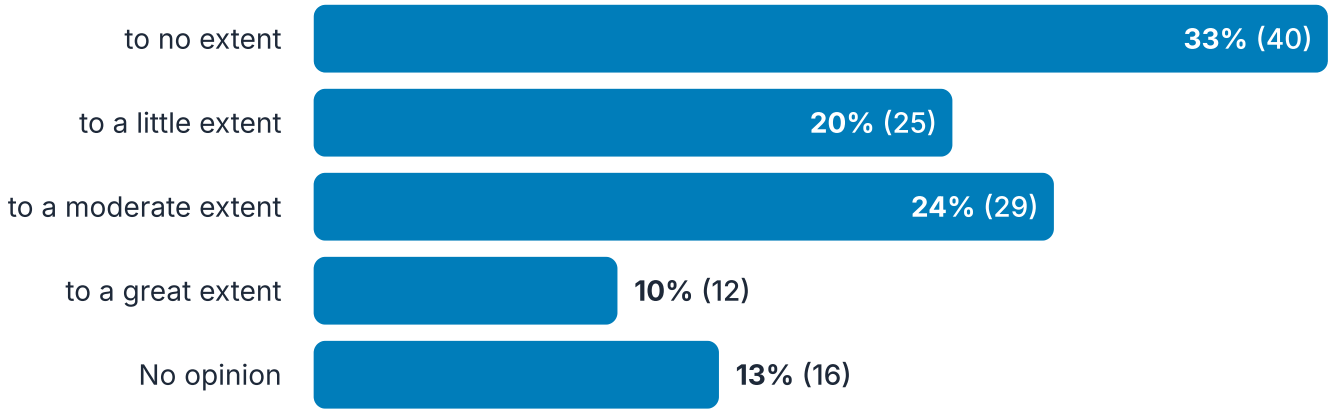


I am unaware of research

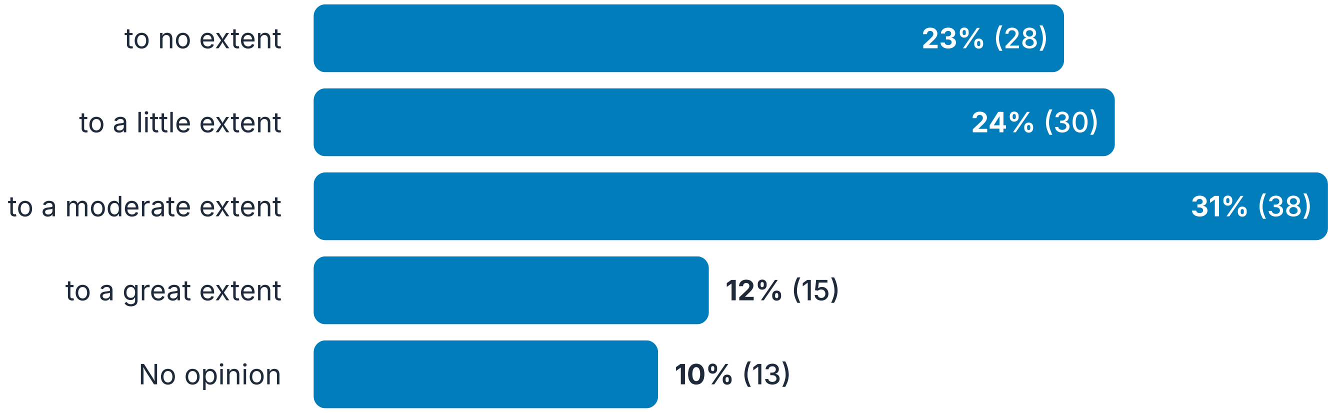


There is a lack of facilities to implement the research

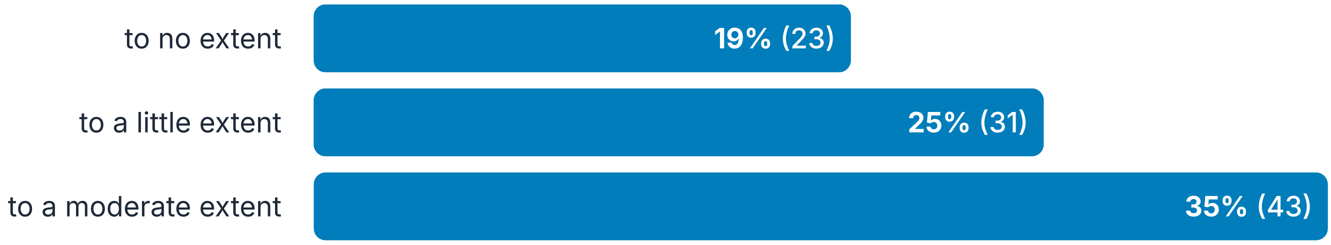

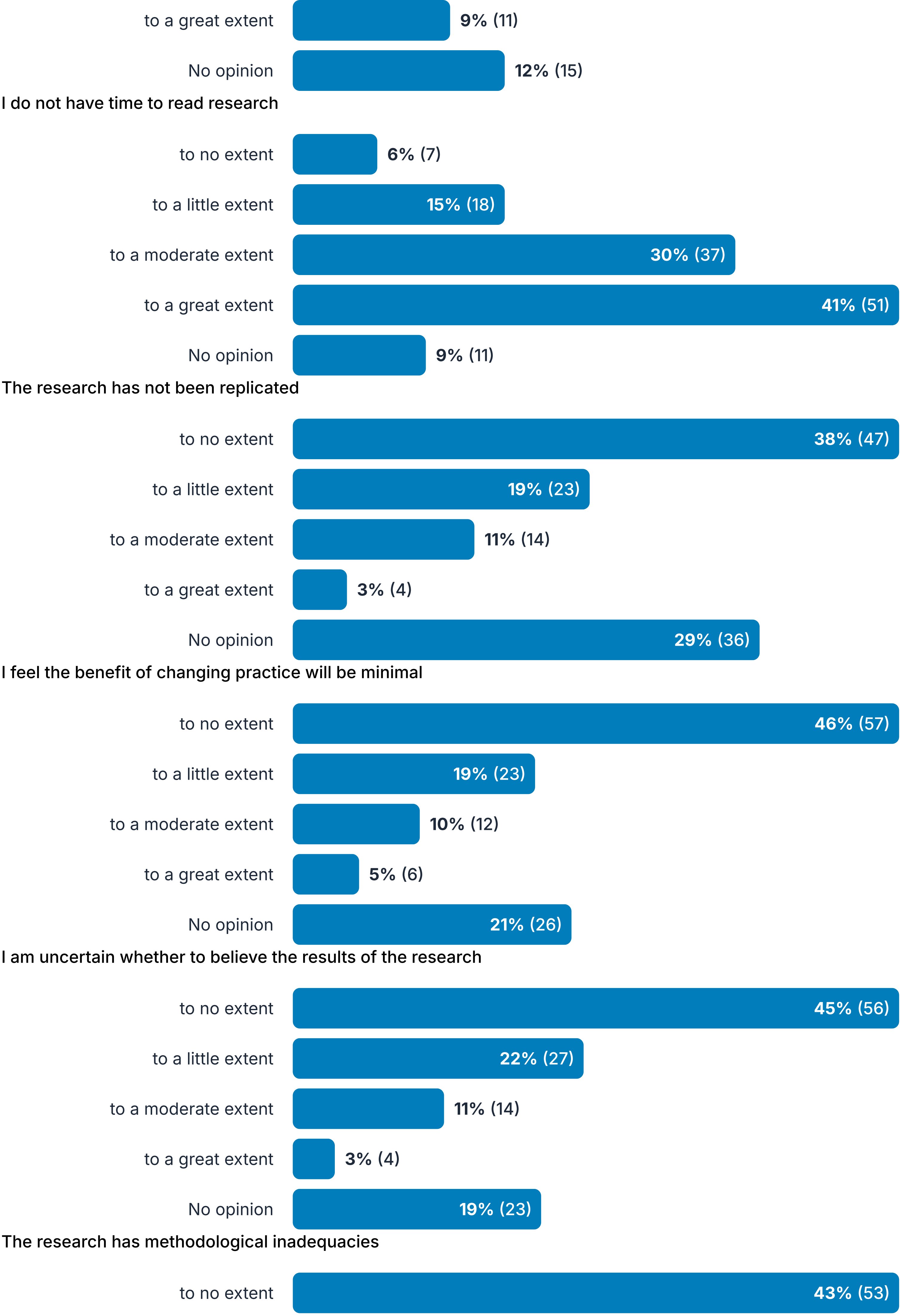
to a great extent
9% (11)
No opinion
12% (15)
I do not have time to read research
to no extent
6% (7)
to a little extent
15% (18)
to a moderate extent
30% (37)
to a great extent
41% (51)
No opinion
9% (11)
The research has not been replicated
to no extent
38% (47)
to a little extent
19% (23)
to a moderate extent
11% (14)
to a great extent
3% (4)
No opinion
29% (36)
I feel the benefit of changing practice will be minimal
to no extent
46% (57)
to a little extent
19% (23)
to a moderate extent
10% (12)
to a great extent
5% (6)
No opinion
21% (26)
I am uncertain whether to believe the results of the research
to no extent
45% (56)
to a little extent
22% (27)
to a moderate extent
11% (14)
to a great extent
3% (4)
No opinion
19% (23)
The research has methodological inadequacies
to no extent
43% (53)

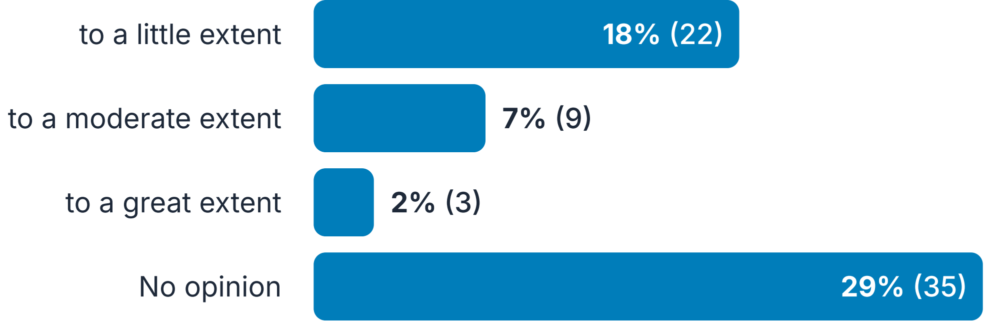


The relevant literature is not compiled in one place

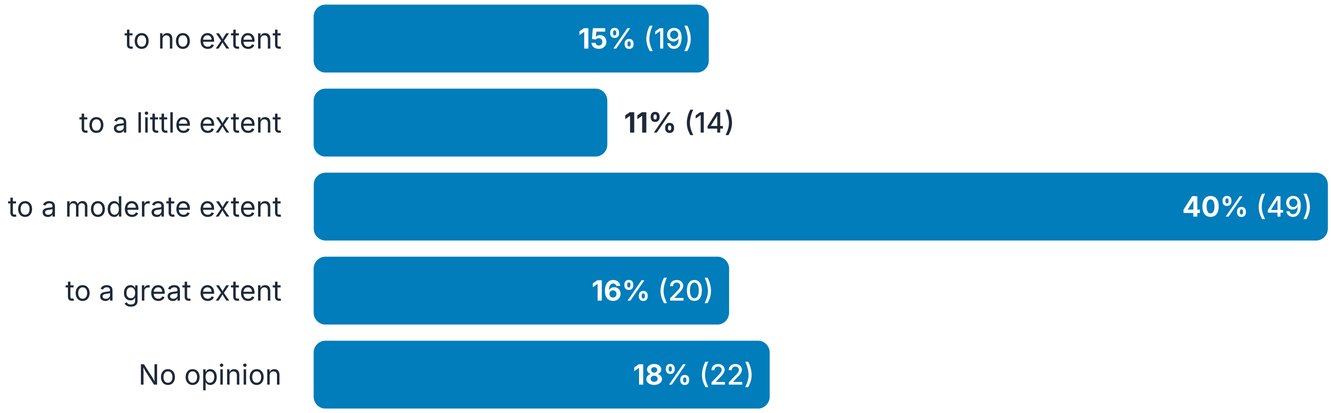


I do not feel I have enough authority to change patient care procedures

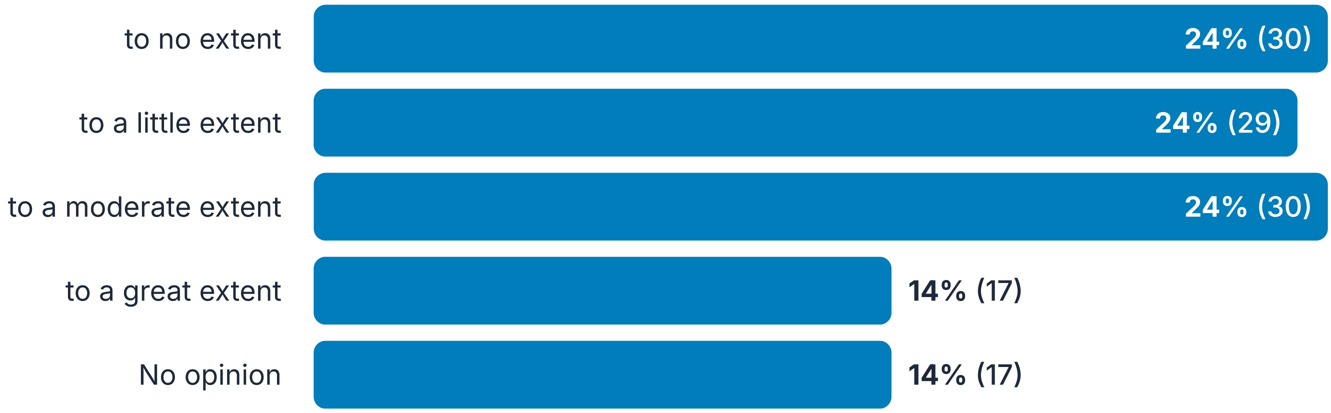


I feel the results are not generalisable to my own setting

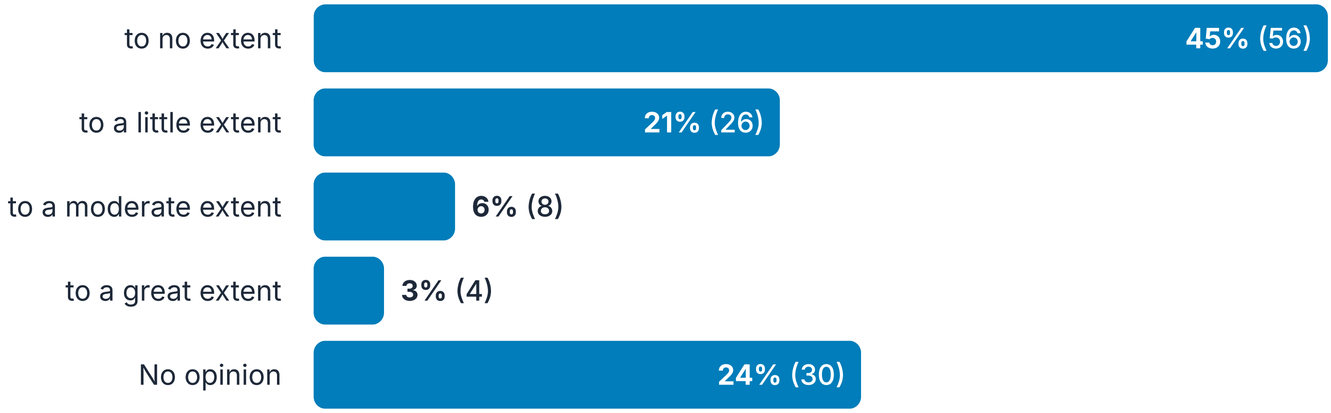


I feel isolated from knowledgeable colleagues with whom to discuss the research

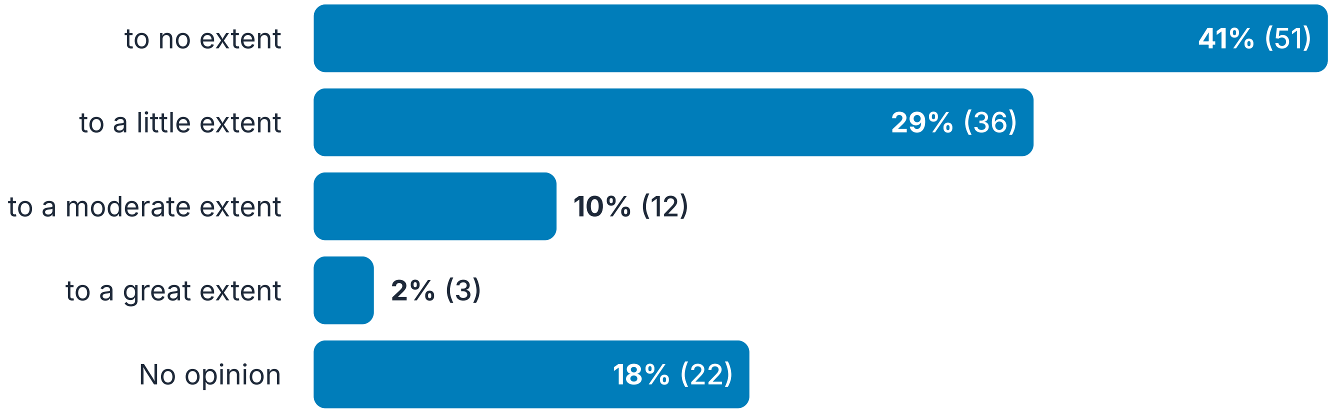

I see little benefit for myself

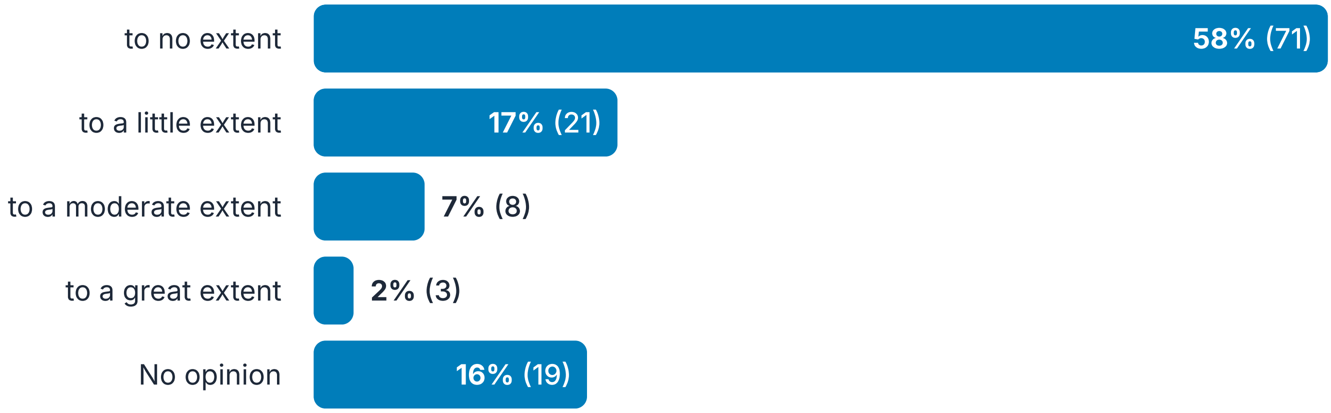


Research reports/articles are not published fast enough

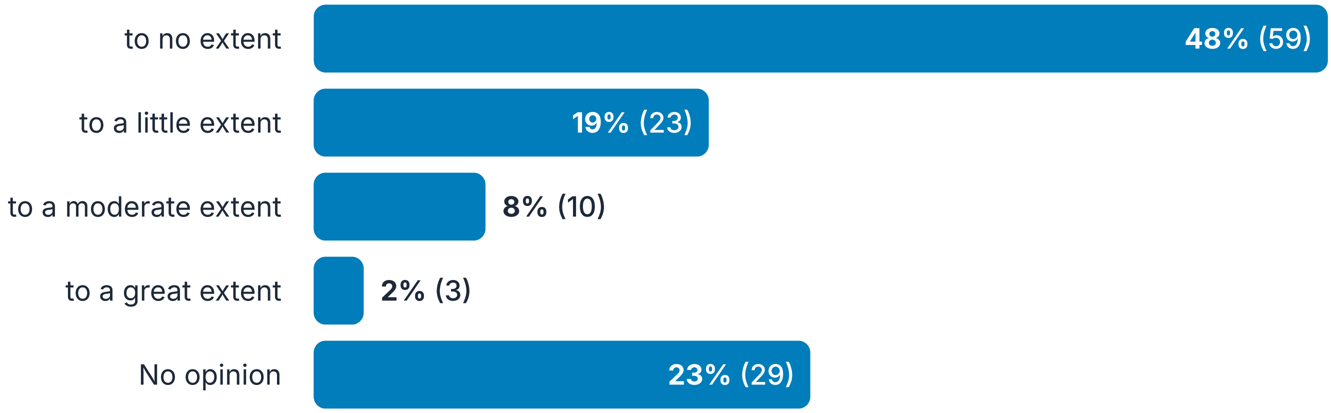


Colleagues will not cooperate with implementation

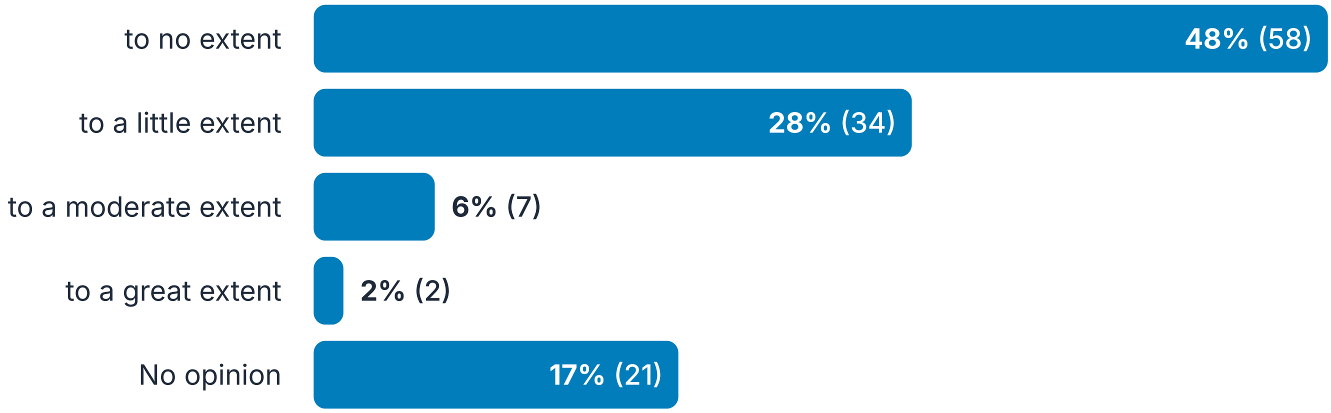


Management will not allow implementation

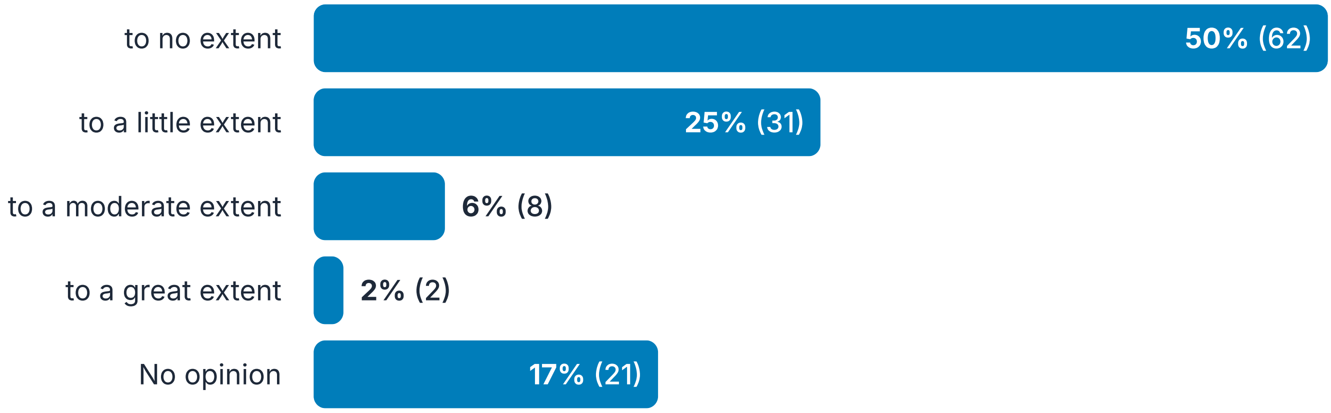


I do not see the value of research for practice

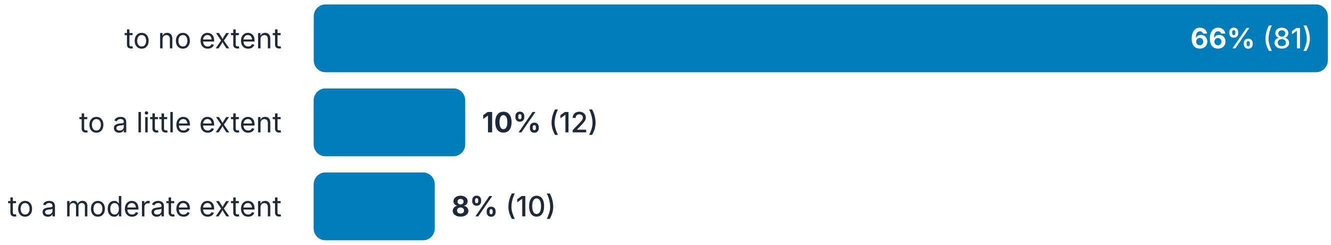

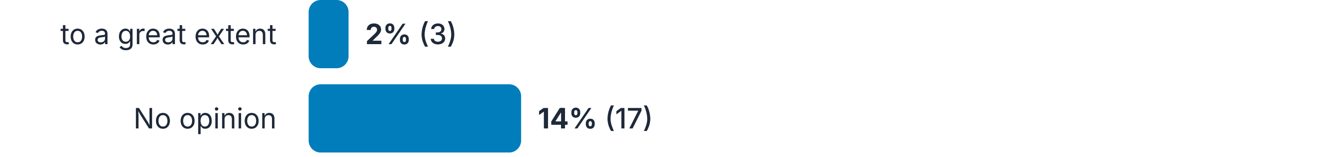


There is not a documented need to change practice

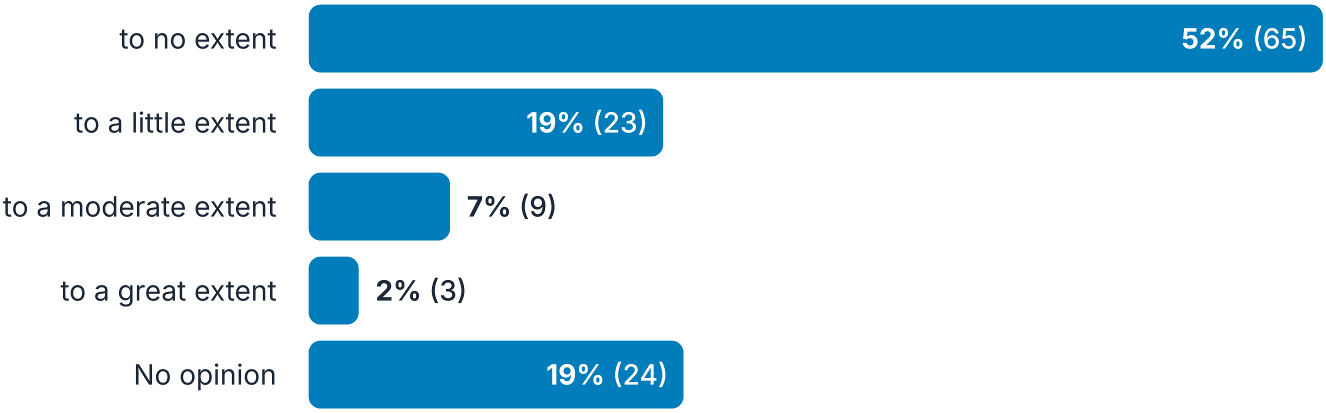


The conclusions drawn from the research are not justified

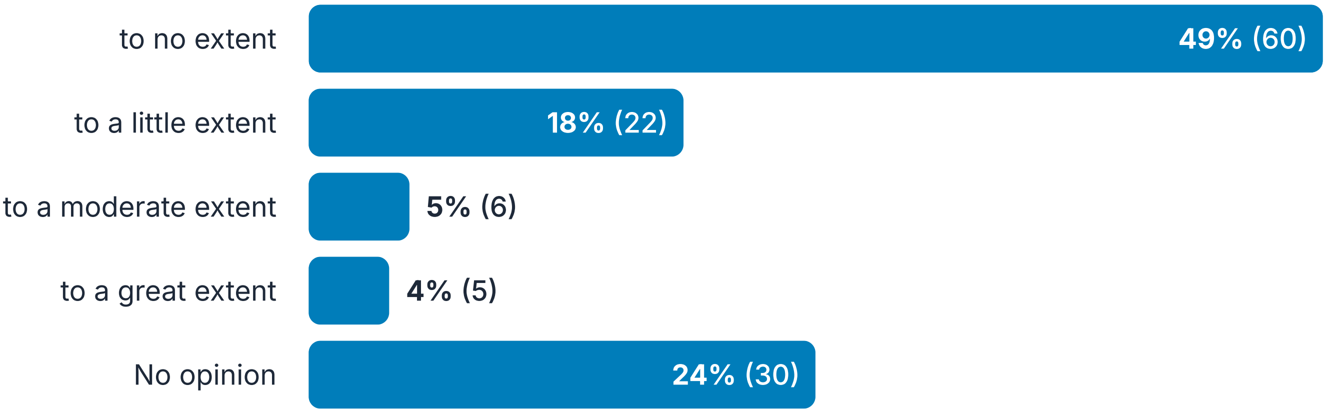


The literature reports conflicting results

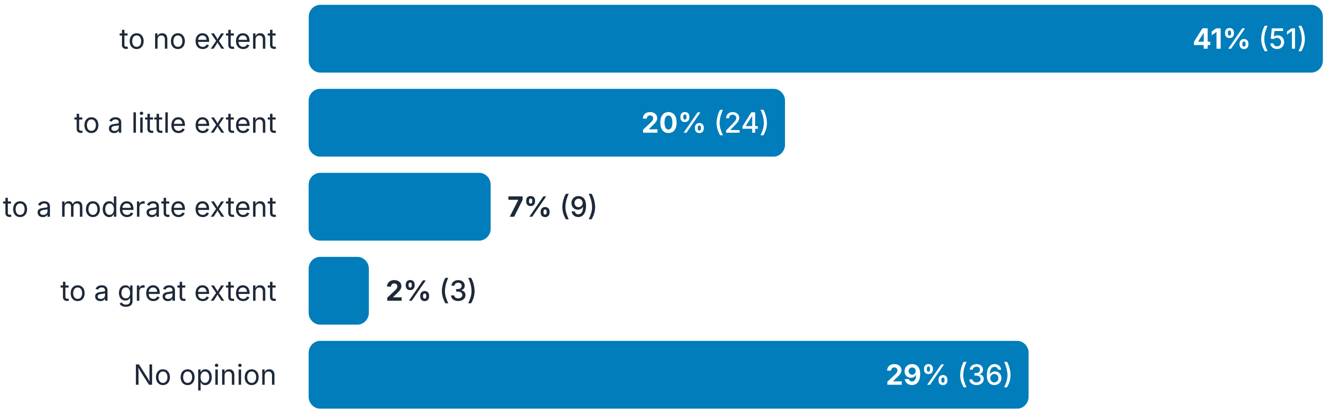


The research is not reported clearly and readably

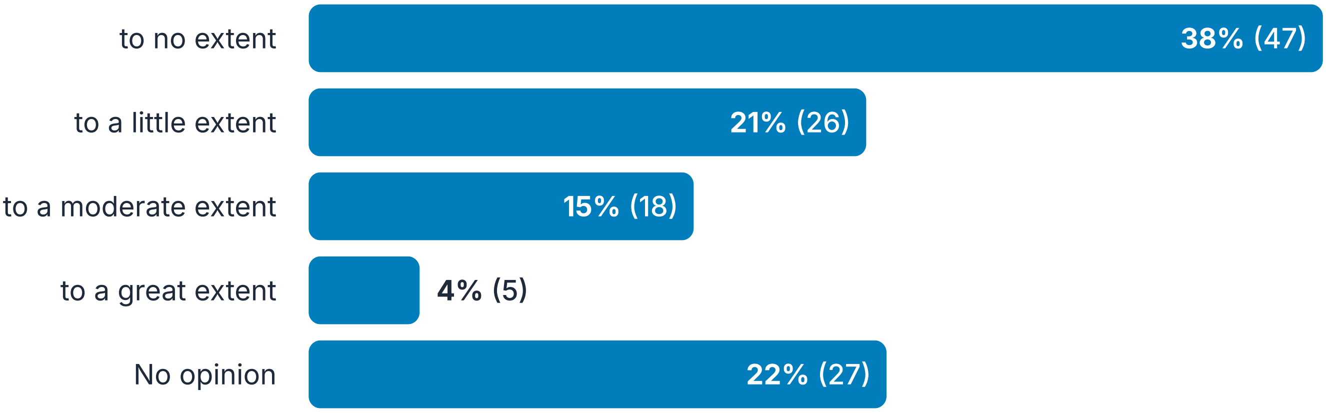


Other staff are not supportive of implementation

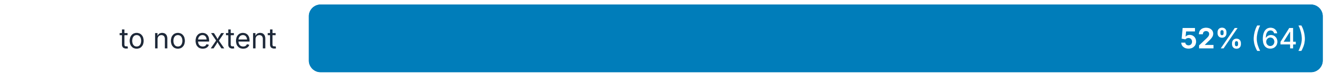

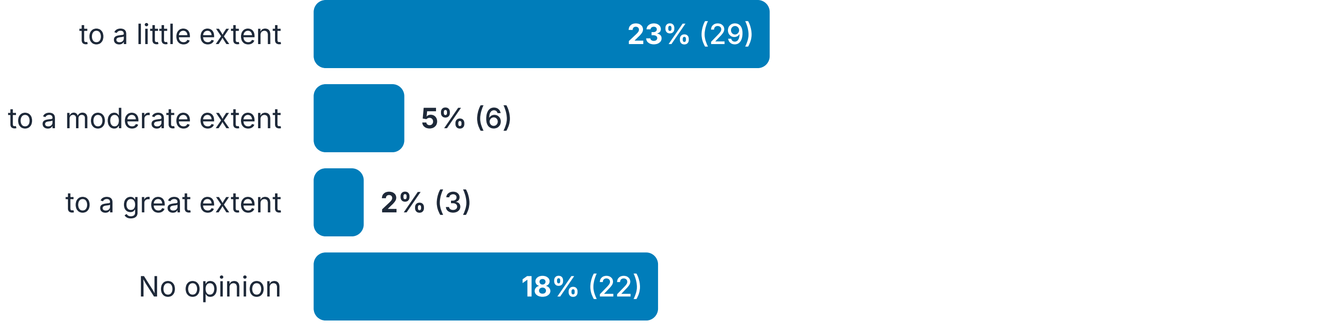


The nurse is unwilling to change/try new ideas

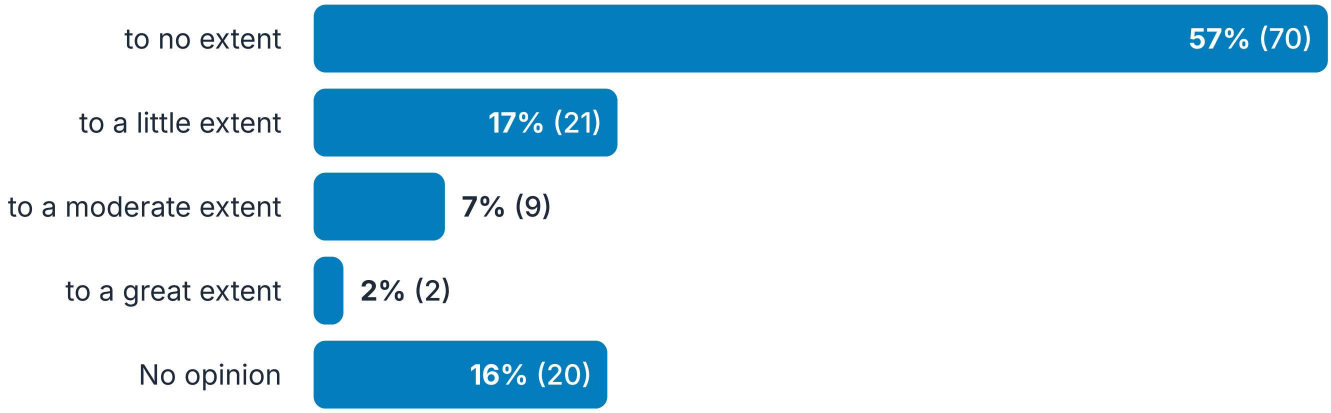


The amount of research information is overwhelming

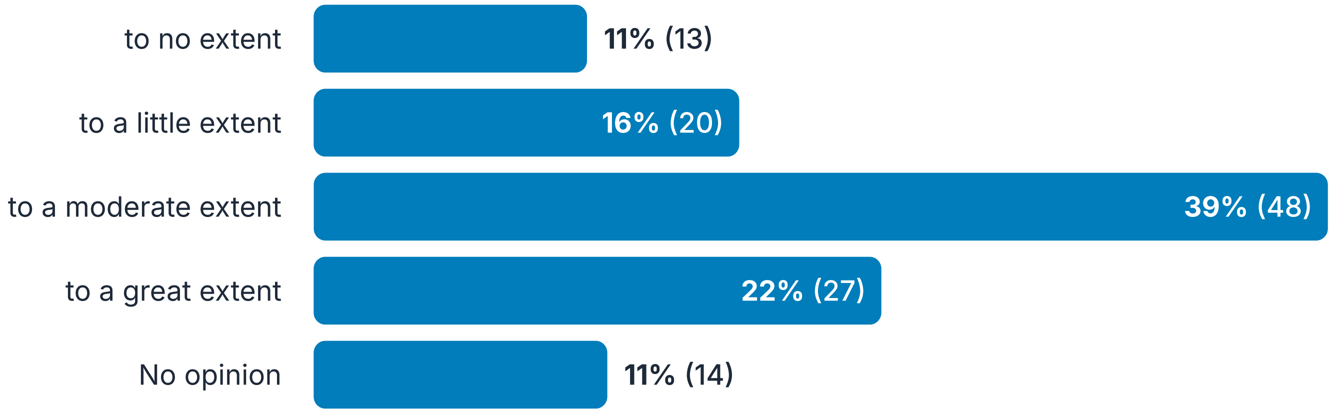


I do not feel capable of evaluating the quality of the research

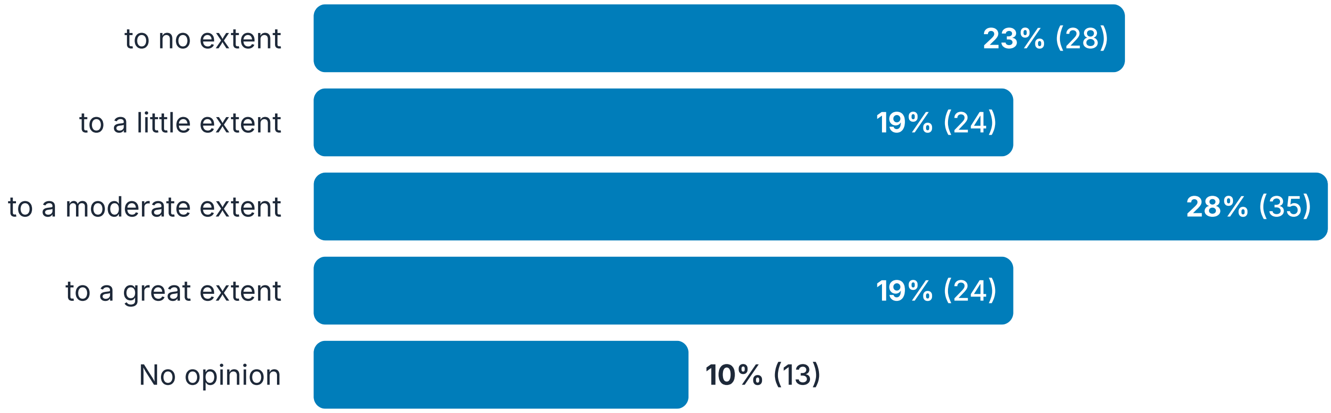


There is insufficient time on the job to implement new ideas

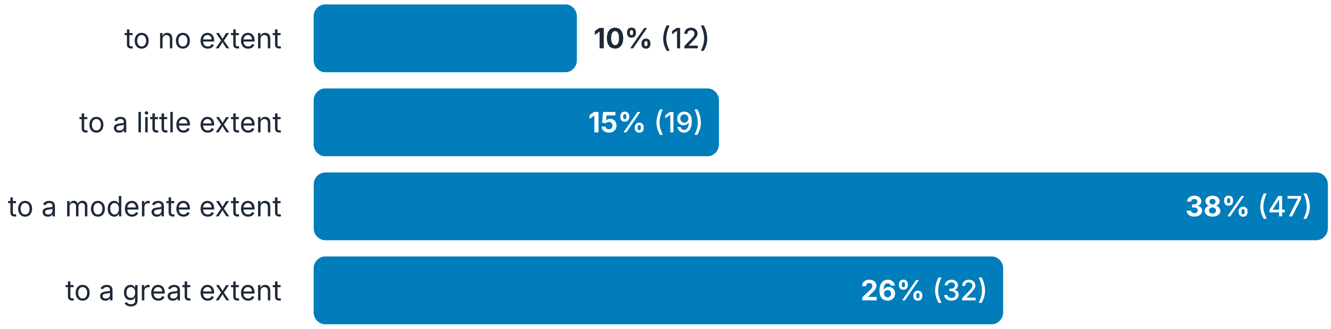

| | |
|---|---|
| No opinion | 11% (13) |

## 21. Overall in your opinion how significant are the overall barriers to utilising evidence based practice in your clinical setting?

Responses: 124

Not significant at all

| | |
|---|---|
| 1 | 6% (7) |
| 2 | 28% (35) |
| 3 | 33% (41) |
| 4 | 33% (41) |

## 22. Using the list below rank what you perceive are the factors that help you use research to inform your clinical practice

Responses: 124

Training/Education days

| | |
|---|---|
| to no extent | 0% (0) |
| to a little extent | 1% (1) |
| to a moderate extent | 17% (21) |
| to a great extent | 76% (94) |
| no opinion | 6% (8) |

Meetings

| | |
|---|---|
| to no extent | 0% (0) |
| to a little extent | 15% (18) |
| to a moderate extent | 31% (37) |
| to a great extent | 48% (57) |
| no opinion | 7% (8) |

Supervision

| | |
|---|---|
| to no extent | 12% (14) |
| to a little extent | 23% (28) |

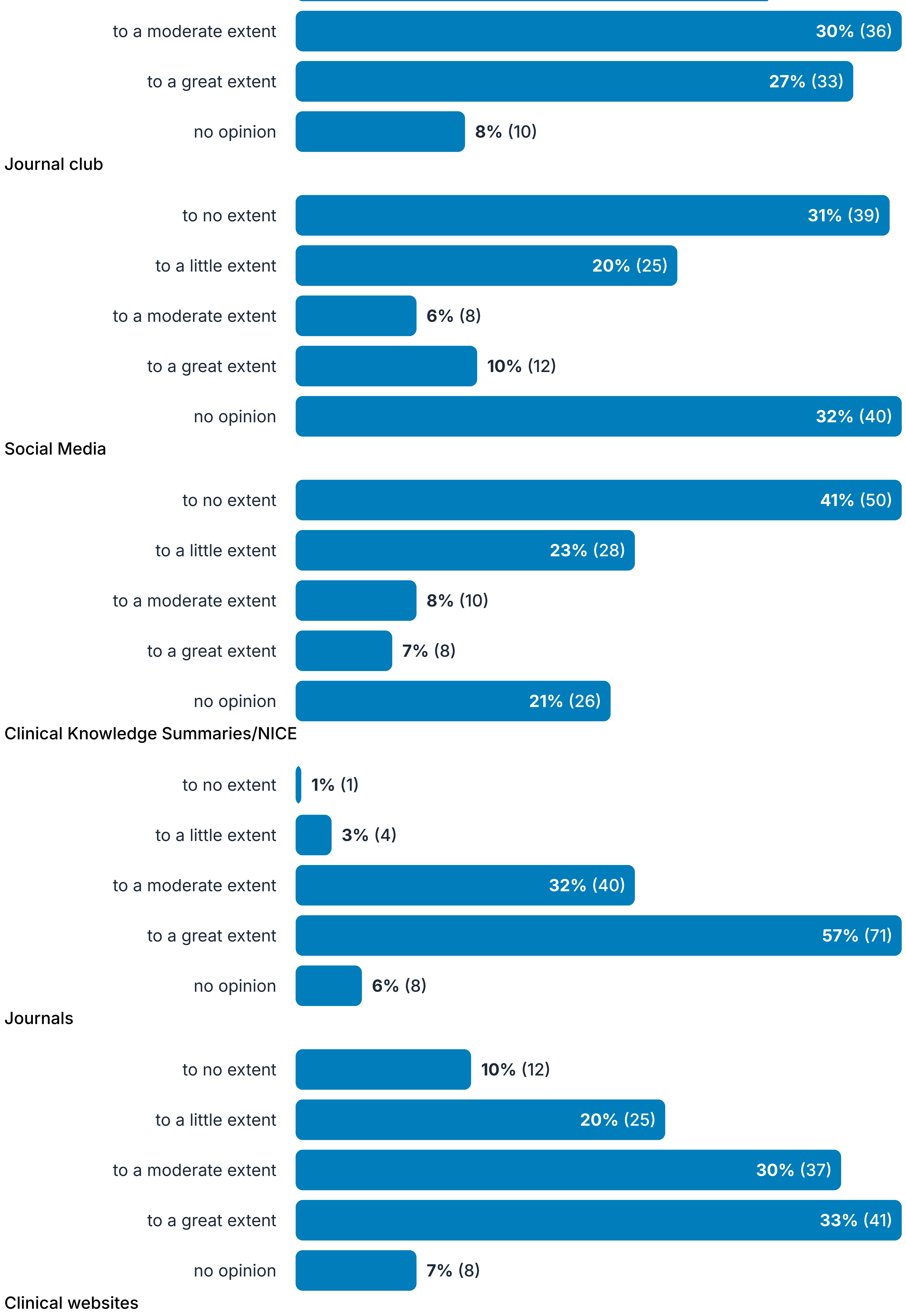
to a moderate extent
30% (36)
to a great extent
27% (33)
no opinion
8% (10)
Journal club
to no extent
31% (39)
to a little extent
20% (25)
to a moderate extent
6% (8)
to a great extent
10% (12)
no opinion
32% (40)
Social Media
to no extent
41% (50)
to a little extent
23% (28)
to a moderate extent
8% (10)
to a great extent
7% (8)
no opinion
21% (26)
Clinical Knowledge Summaries/NICE
to no extent
1% (1)
to a little extent
3% (4)
to a moderate extent
32% (40)
to a great extent
57% (71)
no opinion
6% (8)
Journals
to no extent
10% (12)
to a little extent
20% (25)
to a moderate extent
30% (37)
to a great extent
33% (41)
no opinion
7% (8)
Clinical websites

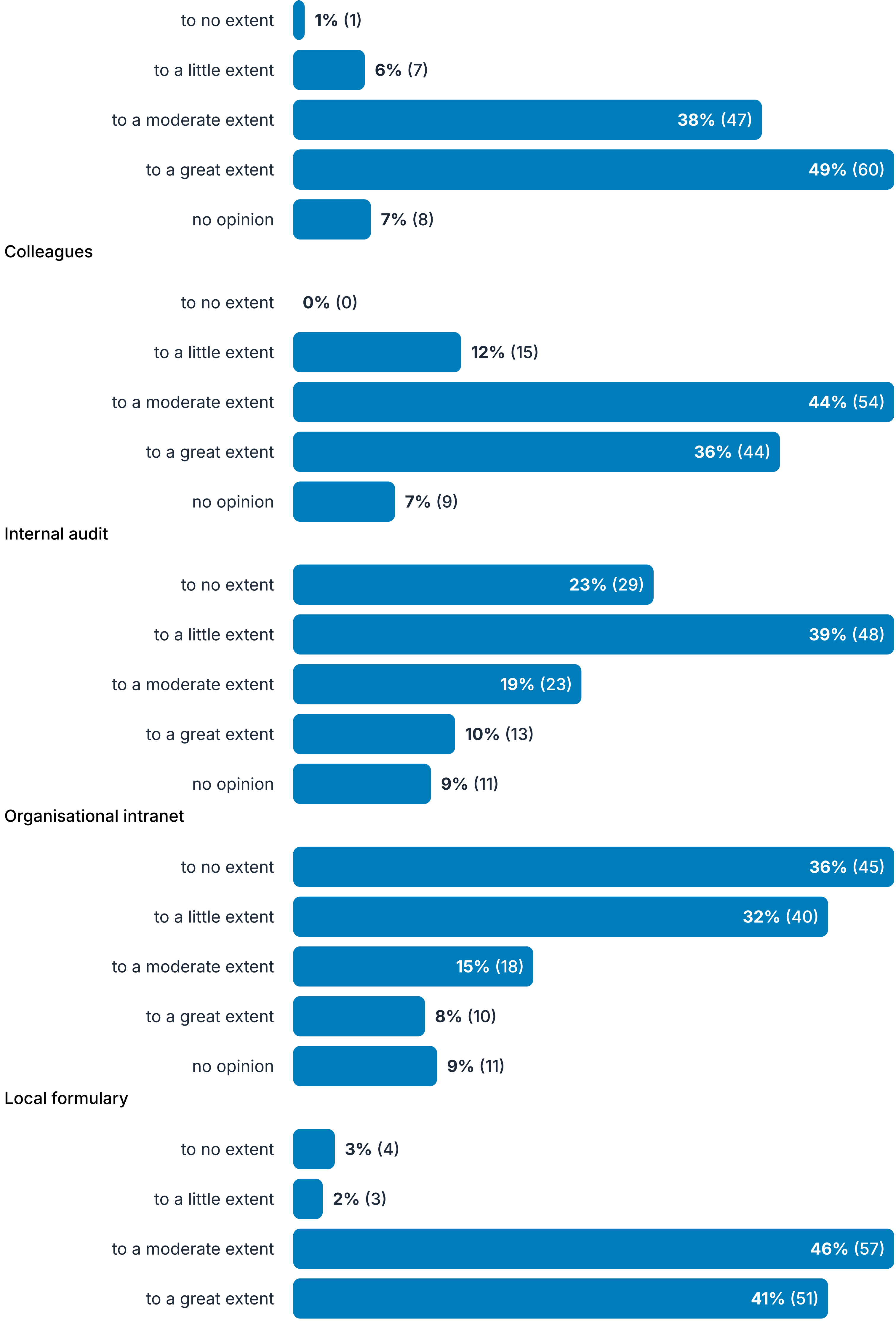

to no extent
1% (1)
to a little extent
6% (7)
to a moderate extent
38% (47)
to a great extent
49% (60)
no opinion
7% (8)
Colleagues
to no extent
0% (0)
to a little extent
12% (15)
to a moderate extent
44% (54)
to a great extent
36% (44)
no opinion
7% (9)
Internal audit
to no extent
23% (29)
to a little extent
39% (48)
to a moderate extent
19% (23)
to a great extent
10% (13)
no opinion
9% (11)
Organisational intranet
to no extent
36% (45)
to a little extent
32% (40)
to a moderate extent
15% (18)
to a great extent
8% (10)
no opinion
9% (11)
Local formulary
to no extent
3% (4)
to a little extent
2% (3)
to a moderate extent
46% (57)
to a great extent
41% (51)

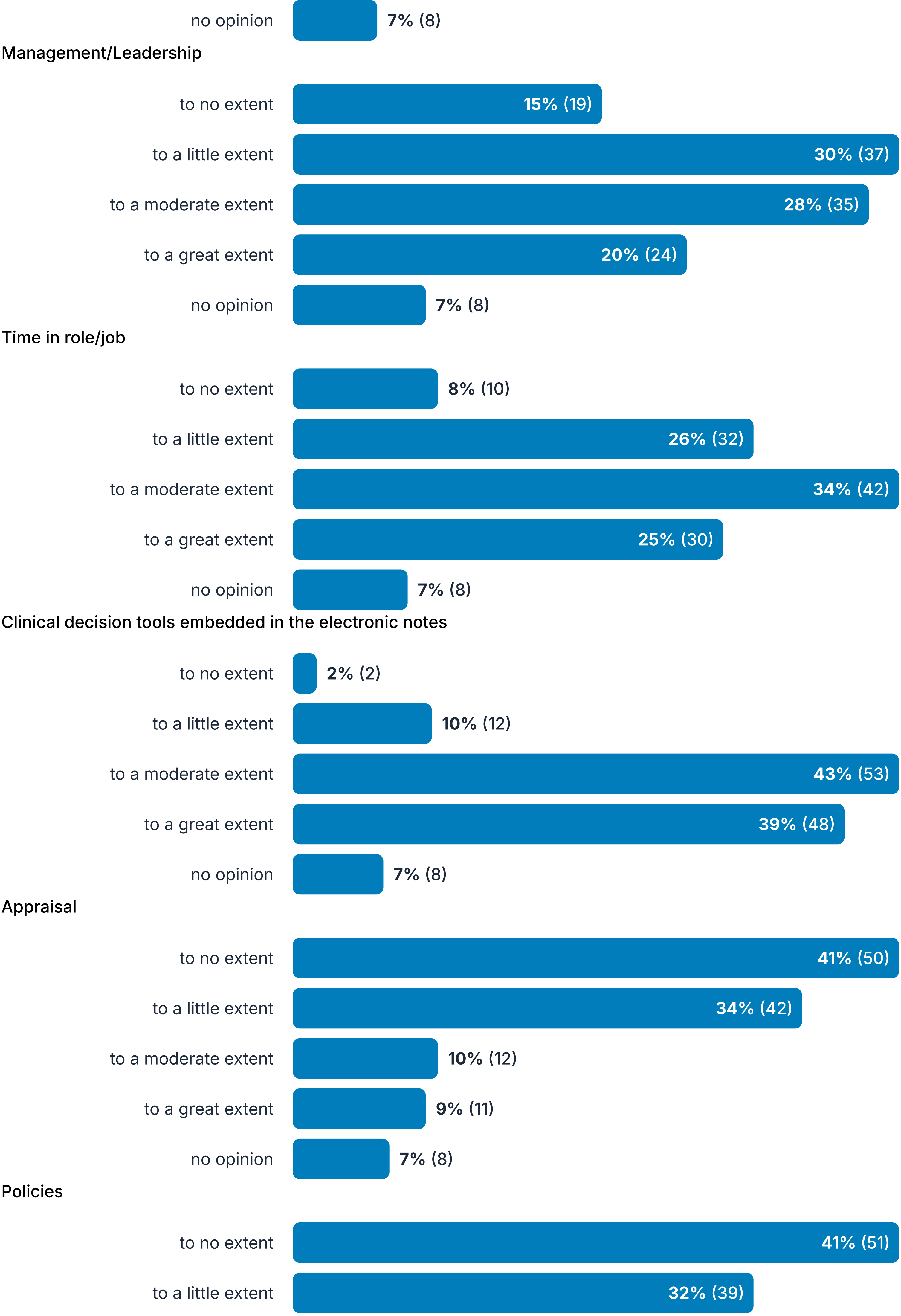
no opinion
7% (8)
Management/Leadership
to no extent
15% (19)
to a little extent
30% (37)
to a moderate extent
28% (35)
to a great extent
20% (24)
no opinion
7% (8)
Time in role/job
to no extent
8% (10)
to a little extent
26% (32)
to a moderate extent
34% (42)
to a great extent
25% (30)
no opinion
7% (8)
Clinical decision tools embedded in the electronic notes
to no extent
2% (2)
to a little extent
10% (12)
to a moderate extent
43% (53)
to a great extent
39% (48)
no opinion
7% (8)
Appraisal
to no extent
41% (50)
to a little extent
34% (42)
to a moderate extent
10% (12)
to a great extent
9% (11)
no opinion
7% (8)
Policies
to no extent
41% (51)
to a little extent
32% (39)

| | |
|---|---|
| to a moderate extent | 10% (12) |
| to a great extent | 11% (13) |
| no opinion | 7% (8) |

Internet search

| | |
|---|---|
| to no extent | 6% (8) |
| to a little extent | 16% (20) |
| to a moderate extent | 39% (48) |
| to a great extent | 32% (40) |
| no opinion | 6% (8) |

Resources to utilise the evidence

| | |
|---|---|
| to no extent | 24% (30) |
| to a little extent | 21% (26) |
| to a moderate extent | 23% (29) |
| to a great extent | 20% (25) |
| no opinion | 11% (14) |

---

## 23. Overall in your opinion how significant are the overall facilitators to utilising evidence based practice in your clinical setting?

Responses: 124

Not significant at all

| | |
|---|---|
| 1 | 3% (4) |
| 2 | 19% (23) |
| 3 | 42% (52) |
| 4 | 36% (45) |
